# Biotransformation and Epithelial Toxicity of Prenylated Phenolics from Licorice Roots (*Glycyrrhiza* spp.) in 3D Apical-Out Mucus-Producing Human Enteroids

**DOI:** 10.1021/acs.jafc.4c03120

**Published:** 2024-09-06

**Authors:** Sarah van Dinteren, Carla Araya-Cloutier, Shanna Bastiaan-Net, Anouk Boudewijn, Tjarda van Heek, Jean-Paul Vincken, Renger Witkamp, Jocelijn Meijerink

**Affiliations:** † Division of Human Nutrition and Health, Wageningen University, P.O. Box 17, Wageningen 6700 AA, The Netherlands; ‡ Laboratory of Food Chemistry, Wageningen University, P.O. Box 17, Wageningen 6700 AA, The Netherlands; § Wageningen Food & Biobased Research, Wageningen University & Research, P.O. Box 17, Wageningen 6700 AA, The Netherlands; ∥ Department of Abdominal Surgery, Hospital Gelderse Vallei, Willy Brandtlaan 10, Ede 6716 RP, The Netherlands

**Keywords:** *Glycyrrhiza glabra*, *G. inflata*, *G. uralensis*, ileal organoids, prenylated phenolics, intestinal models, antimicrobial

## Abstract

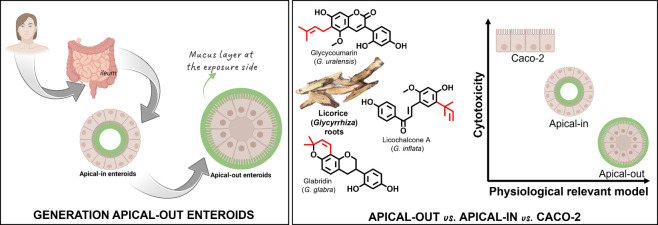

Apical-out enteroids mimic the *in vivo* environment well due to their accessible apical surface and mucus layer, making them an ideal model for studying the impact of (bioactive) food compounds. Generated human ileal apical-out enteroids showed a fucose-containing mucus layer surrounding the apical brush border on their exposure side, indicating their physiological relevance. Effects on the mucosal epithelium of antibacterial prenylated phenolics (glabridin, licochalcone A, and glycycoumarin) from licorice roots were investigated for cytotoxicity, cell viability, barrier integrity, and biotransformation. At concentrations up to 500 μg mL^–1^, licochalcone A and glycycoumarin did not significantly affect apical-out enteroids, with cytotoxicities of −6 ± 2 and −2 ± 2% and cell viabilities of 77 ± 22 and 77 ± 13%, respectively (*p* > 0.05). Conversely, 500 μg mL^–1^ glabridin induced significant cytotoxicity (31 ± 25%, *p* < 0.05) and reduced cell viability (21 ± 14%, *p* < 0.01). Apical-out enteroids revealed differential sensitivities to prenylated phenolics not observed in apical-in enteroids and Caco-2 cells. Both enteroid models showed phase II biotransformation but differed in the extent of glucuronide conversion. The apical mucus layer of apical-out enteroids likely contributed to these differential interactions, potentially due to differences in electrostatic repulsion. This study underscores the relevance of 3D apical-out enteroid models and highlights the promise of prenylated phenolics for antimicrobial applications.

## 1 Introduction

The intestinal epithelium shows high plasticity and performs dual functions: it serves as a physical barrier, preventing the entry of pathogens and toxic compounds from the external environment while at the same time allowing the absorption of nutrients. The epithelial cell layer consists of four major differentiated cell types that all derive from adult stem cells that are located at the crypt bottoms, including (1) absorptive enterocytes, (2) mucus secreting goblet cells, (3) hormone-producing enteroendocrine cells, and (4) Paneth cells that secrete antimicrobial peptides.^1^ The epithelium of the small intestine is covered with a single layer of loosely, unattached, viscous, gel-forming, highly glycosylated (e.g., glucose, *N-*acetylglucosamine, and fucose) mucins, termed mucus (formed by MUC2). Mucus is paramount for protection^2,3^ and lubrication^4,5^ and can entrap nutrients and xenobiotics, present in the gastrointestinal tract.^6^ Traditionally, the *in vitro* assessment of gut–nutrient interactions, intestinal physiology, diseases, and cytotoxicity has relied on the use of intestinal cell lines. Advantages of using cell culture systems include target-restricted experimentation, high consistency, and high reproducibility.^7^ However, cell cultures often lack the complexity of their *in vivo* counterpart. For example, the often used differentiated Caco-2 cells, considered representative of the small intestine but originating from a colon tumor, contain predominantly absorptive enterocytes, do not contain a proper mucus layer, and exhibit various genotypic and phenotypic functional aberrations.^8−11^

Intestinal 3D organoids are a relatively new *in vitro* model that recapitulates the *in vivo* intestinal epithelium. They maintain the basic crypt-villus morphology of the intestine and are composed of the different epithelial cell types and their reciprocal interactions.^12^ Conventionally, organoids are grown in a 3D extracellular protein matrix (ECM) surrounded by a growth medium with appropriate growth factors. In this model, the apical or luminal surface is facing the organoids’ interior (apical-in organoids),^12^ making this model less appealing for nutritional, microbial, or physiological studies.^13^ In order to study epithelial interactions with luminal contents, an organoid cultivation technique was recently developed that maintains the 3D organoid structure and functions in a suspension while making the apical surface accessible to experimental challenges (apical-out organoids).^13,14^ We recently showed that apical-out small intestinal mouse organoids (enteroids) provide a more accurate representation of the *in vivo* environment, among others reflected by the presence of an apical mucus layer on the exposed side.^15^ Additionally, we showed that mouse apical-out enteroids better represent the small intestine than apical-in enteroids, as shown by comparisons of gene expression of epithelial cell markers (e.g., for stem cells, enterocytes, goblet cells, enteroendocrine cells, and Paneth cells) with those from the *in vivo* tissue derived from the same location.^15,16^

**Figure 1:**
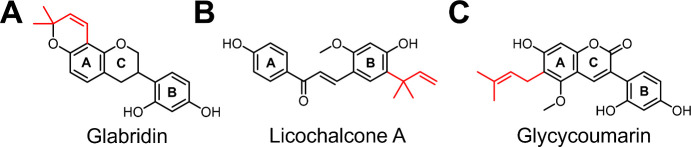
Species-specific (iso)flavonoids glabridin (glab), licochalcone A (licoA), and glycycoumarin (glycy). Molecular structures of the main species-specific compounds glab (ring-prenylated isoflavan from *G. glabra*), licoA (chain-prenylated chalcone from *G. inflata*), and glycy (chain-prenylated 3-arylcoumarin from *G. uralensis*). The prenyl group (indicated in red) is largely contributing to its antibacterial activity. Adapted with permission from ref (15). Copyright 2024 Royal Society of Chemistry.

We have recently demonstrated promising antibacterial activity of prenylated (iso)flavonoids and chalcones (phenolics) found in the prenylated phenolic-rich waste streams of licorice (*Glycyrrhiza* spp.) roots.^17^ Prenylation of phenolics generally increases antibacterial activity due to enhanced interaction and/or disruption of bacterial membranes.^18,19^ Particularly, the licorice-specific compounds glabridin (glab, from *Glycyrrhiza glabra* (*G. glabra*)), licochalcone A (licoA, from *Glycyrrhiza inflata* (*G. inflata*)), and glycycoumarin (glycy, from *Glycyrrhiza uralensis* (*G. uralensis*)) displayed significant antibacterial activity against various Gram-positive bacteria (Figure 1). With minimum inhibitory concentrations (i.e., MIC) ranging between 3.1 and 25 μg mL^–1^, these compounds hold promise for combating food spoilage and pathogens such as *Lactobacillus buchneri* (*L. buchneri*) and *Staphylococcus aureus* (*S. aureus*).^17^ However, their high affinity for bacterial membranes raises concerns about their potential interaction with the gastrointestinal epithelium.^18^

In this study, we investigated how three structurally related prenylated phenolics, glab, licoA, and glycy from licorice roots, interact with and possibly affect the intestinal epithelium. For this, we studied their effects on cytotoxicity, cell viability, biotransformation, and barrier integrity in an innovative and physiologically relevant human 3D apical-out enteroid model. Additionally, we compared these outcomes to the conventional 3D apical-in enteroid model and placed the results from apical-out and apical-in enteroids from this study with human enteroids in context with those previously obtained for mouse enteroids^15^ and Caco-2 cells.^17^

## 2 Materials and Methods

### 2.1 Materials

Glabridin (glab) (≥97.0%) was purchased from Wako (Osaka, Japan); licochalcone A (licoA) (≥96.0%), Triton X-100, and FITC-dextran 4 kDa were purchased from Sigma-Aldrich (St. Louis, MO, USA); glycycoumarin (glycy) (≥98.0%) was purchased from ChemFaces (Wuhan, China; confirmed by RP-UHPLC-PDA-MS^n^); phosphate-buffered saline (PBS), Dulbecco’s modified Eagle’s medium supplemented with glucose (4.5 g L^–1^) (DMEM), HEPES buffer solution (0.58 g L^–1^), l-glutamine (0.58 g L^–1^), 10% (v/v) heat-inactivated fetal calf serum (FCS), and 1% (v/v) penicillin and streptomycin, methanol-free formaldehyde, Tween-20, and GlutaMAX supplement were purchased from Thermo Fisher Scientific Gibco (Waltham, Massachusetts, USA); DMEM/F12 with a 15 mM HEPES buffer, gentle cell dissociation reagent (GCDR), anti-adherence solution, IntestiCult human organoid growth medium (OGM), IntestiCult human differentiation medium (ODM), *N*-[2*S*-(3,5-difluorophenyl)acetyl]-l-alanyl-2-phenyl-1,1-dimethylethylester glycine (DAPT), and bovine serum albumin were purchased from STEMCELL Technologies (Vancouver, Canada); Matrigel growth factor-reduced basement membrane matrix phenol red-free (MG) and penicillin/streptomycin (P/S) solution 100× were purchased from Corning Incorporated (Somerville, Massachusetts, USA). Acetonitrile (ACN) and water acidified with 0.1% (v/v) formic acid (FA) were purchased from Biosolve (Valkenswaard, The Netherlands); dimethyl sulfoxide (DMSO) was purchased from Merck Millipore (Billerica, MA, USA). Water for purposes other than UHPLC was prepared by using a Milli-Q water purification system (Merck Millipore).

### 2.2 Generation of 3D Apical-In Ileal Enteroids

Research on human organoids was set up through collaboration between Wageningen University & Research and the Hospital Gelderse Vallei (ZGV). Human ileal enteroids were generated from the ileal tissue obtained from a female donor (age, 80 years; BMI, 21.1 kg/m^2^) during surgery. The regional Medical Ethical Review Committee (METC) decided that no approval was required, as the ileal tissue constituted residual body material and was no longer required for patient care, aligning with Dutch legislation. Subsequently, the research protocol was reviewed and received approval from the local METC of the ZGV. Prior to surgery, the patient was provided with an information brochure, granted more than a week for reflection, and provided informed consent. Enteroids keep the same characteristics of the location where they are derived from, in this case the ileum.^17,20,21^ Frozen ileal enteroids (∼200 enteroids/cryovial) were thawed at 37 °C and mixed with DMEM/F12 supplemented with 1% (w/v) BSA, 15 mM HEPES buffer solution, GlutaMAX supplement, and 1% (v/v) P/S (abbreviated as DMEM/F12). An enteroid suspension was centrifuged at 200*g* for 5 min at 4 °C, after which the pellet was 1:1 mixed with MG and DMEM/F12. Domes of 50 μL were plated on a prewarmed 24-well plate. Per well, 500 μL of OGM was added. The medium was changed every 2 days. For passaging, enteroids were fully grown in an OGM and passaged every 8–14 days in a 1:3 to 1:4 split ratio.

### 2.3 Generation of 3D Apical-Out Ileal Enteroids

Apical-out ileal enteroids were generated, as described elsewhere.^13−15^ Briefly, the medium was aspirated from ∼200 apical-in enteroids (1 dome) at day 7–10 grown in OGM, and MG containing the enteroids was removed with GCDR and incubated (in prewetted tubes with anti-adherence solution) for 40 min at 4 °C with continuous agitation. Enteroids were pelleted by centrifugation at 250*g* for 3 min at 4 °C and were washed three times with DMEM/F12 to remove MG. To induce differentiation, enteroids were resuspended in ODM supplemented with 5 μM DAPT (hereafter abbreviated as ODM) in ultralow binding culture plates (Corning) and incubated at 37 °C with 5% CO_2_. The morphology of the enteroids was observed daily under a microscope to check polarity reversal (Section 2.5). ODM was changed every 2 days, and enteroids were passed from apical-in to apical-out in a 1:2 split ratio. Full polarity reversal was observed 72 h after MG removal.

### 2.4 Caco-2 Cell Culture

**Figure 2:**
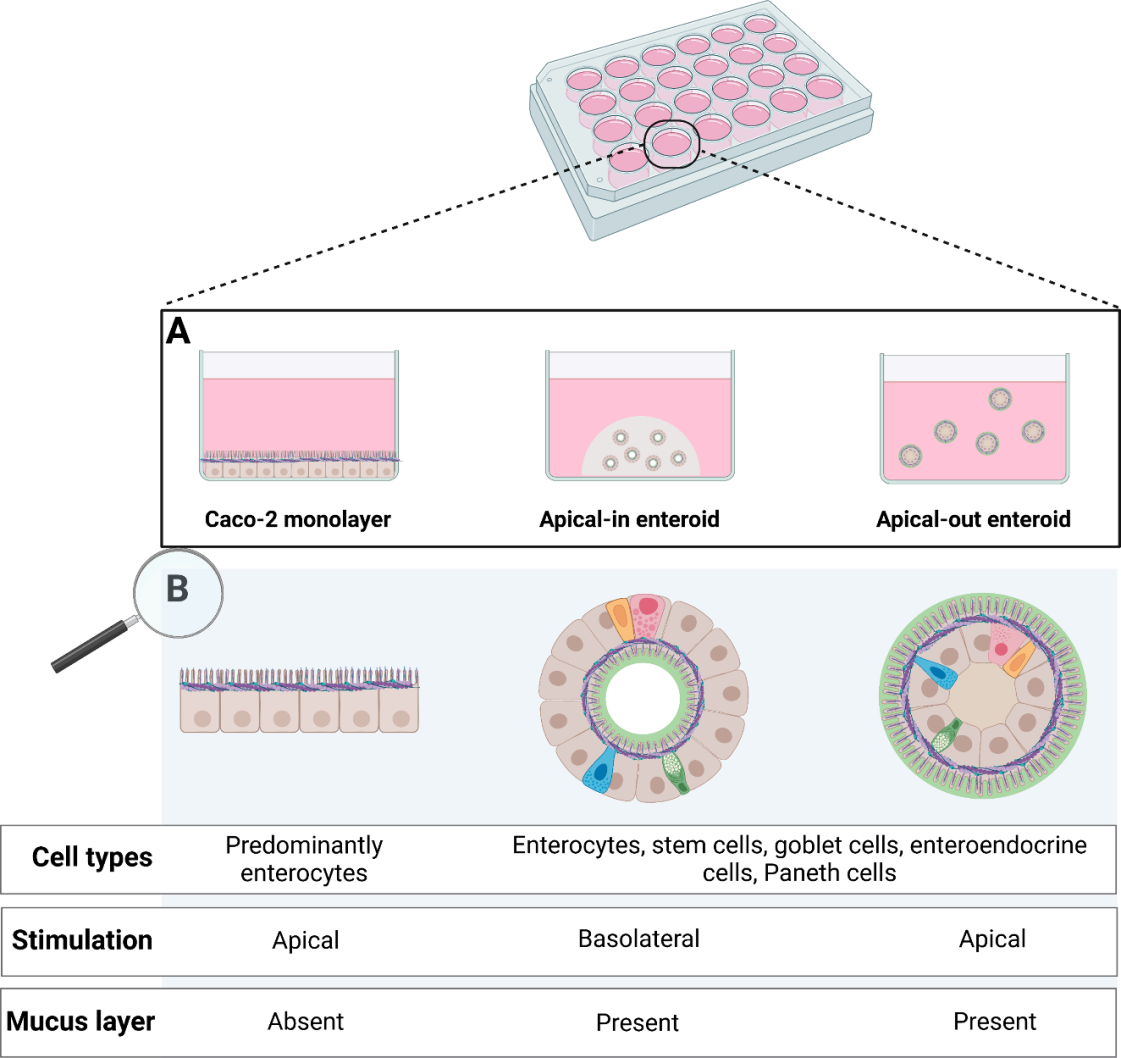
Overview of the different *in vitro* models to determine cytotoxicity and cell viability of prenylated phenolics.^15,17^ Panel (A) shows a schematic overview of the Caco-2 cell monolayer, apical-in enteroids embedded in an extracellular protein scaffold (ECM), and apical-out enteroids suspended in a culture medium. Panel (B) shows a schematic magnified view of the different models: absorptive enterocytes with nuclei are shown in light brown, stem cells are shown in red, Paneth cells are shown in orange, enteroendocrine cells are shown in blue, and goblet cells are shown in green. The apical microvilli brush border is shown in purple, and the apical mucus layer is shown as a dark green layer in apical-in and apical-out enteroids. This figure was created with BioRender.com.

The human colon adenocarcinoma cell line (Caco-2) was obtained from the American Type Culture Collection (ATCC; Manassas, VA, USA). Caco-2 cells were cultured in DMEM, in a humidified atmosphere of 5% CO_2_ at 37 °C until 80–90% confluency was reached. Effects on the cytotoxicity and cell viability (Section 2.6) were assessed in proliferating and differentiated Caco-2 cells. The effects on differentiated Caco-2 cells were recently described in our publication.^17^ Proliferating and differentiated Caco-2 cells were seeded into 96-well plates at a density of 15,000 cells per cm^2^. An overview of the different *in vitro* models used in this study is shown in Figure 2.^15,17^

### 2.5 Evaluation of Polarity Reversal in Ileal Enteroids

Ileal enteroids were collected (directly after MG removal and subsequently at *t* = 0, 24, 48, and 72 h after polarity reversal) and fixed with 3.7% MeOH-free paraformaldehyde solution in PBS for 45 min at room temperature (RT). Enteroids were subsequently permeabilized and blocked with 5% (w/v) BSA and 2% (v/v) Triton X-100 for 60 min. F-actin in the microvilli brush border was stained with ActinRed 555 (2 drops mL^–1^, ReadyProbes Reagent, Invitrogen, Waltham, MA, United States) or Alexa Fluor 660 phallaoidin (1:400, Invitrogen), fucose units in the mucus layer with *Ulex europaeus* agglutinin I conjugated to rhodamine (UEA-1) (1:100, Vector Laboratories, CA, United States), and nuclei with DAPI dilactate (1500 nM, Invitrogen), according to the manufacturer’s instructions.

In brief, fixed and permeabilized enteroids were washed three times with an immunofluorescence buffer (PBS with 0.1% (w/v) BSA, 0.2% (v/v) Triton X-100, and 0.1% (v/v) Tween-20) (centrifuged at 200*g* for 2 min at RT) and subsequently stained with F-actin and incubated for 25 min followed by UEA-1 staining for 30–60 min and 15 min nuclei staining with DAPI for 15 min.

All centrifugation steps were performed at RT. Enteroids were washed and resuspended in PBS, after which they were imaged with an EVOS FL Auto 2 cell imaging system (Invitrogen). For confocal microscopy, stained enteroids were transferred to a chambered glass coverslip (Ibidi, Gräfelfing, Germany), after which they were imaged with a rescan confocal microscope (RCM1, confocal.nl, Amsterdam, The Netherlands). Images were analyzed by using ImageJ software (version 1.52).

### 2.6 Enteroid and Caco-2 Exposure to Glabridin, Licochalcone A, and Glycycoumarin

Cytotoxicity and effects on cell viability after exposure to glab, licoA, and glycy in apical-out and apical-in enteroids (6.25–500 μg mL^–1^) and proliferating Caco-2 cells (3.13–100 μg mL^–1^) were determined by LDH leakage (Section 2.6.1) and WST-1 assays (Section 2.6.2), respectively. For this, stock solutions of 50 mg mL^–1^ of glab, licoA, and glycy in DMSO were used. The highest concentration of DMSO in the measurement was 1% (v/v), which did not yield signs of cytotoxicity or effects on cell viability markers. An overview of the experimental conditions for the exposure experiments with glab, licoA, and glycy is shown in Table S2 (Supporting Information). In brief, apical-out and apical-in enteroids were grown for 7–10 days in OGM (∼200 enteroids per dome), after which the medium was changed to ODM (supplemented with 5 μM DAPT) to initiate enteroid differentiation. To generate apical-out enteroids, MG was removed from apical-in enteroids in OGM at day 7–10, as described in Section 2.3, and suspended in supplemented ODM. After 3 days in ODM, the effects on cytotoxicity and cell viability of glab, licoA, and glycy in apical-out and apical-in enteroids (passages between 3 and 17) were assessed after 4 and 24 h of incubation. For apical-out enteroids in a suspension, enteroids were pelleted by centrifugation (2 min, 200*g*, RT), after which the ODM was removed and 500 μL of the experimental agent was added. Apical-in enteroids in MG did not require centrifugation, and the experimental agent was added after removing the ODM. Caco-2 cells were grown for 48 h after seeding before experiments (passages 14–19) with proliferating cells and for 21 days after seeding (passages 14–33) with differentiated cells.^17^ The medium (DMEM supplemented with FCS) was changed every 2 days. For exposure experiments, Caco-2 cells were incubated with glab, licoA, and glycy in DMEM without FCS, as FCS serves as an exogenous source of LDH.^22^

#### 2.6.1 Cytotoxicity Assessed by the LDH Leakage Assay

Cytotoxic effects of glab, licoA, and glycy were assessed by measuring leakage of intracellular lactate dehydrogenase (LDH) in the supernatant and analyzed using an LDH cytotoxicity detection kit (Roche Applied Science, Almere, The Netherlands), according to the manufacturer’s instructions. The LDH activity in the supernatant was expressed as the percentage of the maximum releasable LDH in enteroids or Caco-2 cells (enteroids or Caco-2 cells treated with 1% (v/v) Triton X-100) and calculated with eq 1.
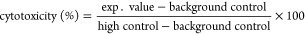
1in which exp. value is the UV absorbance at 492 nm (Tecan Spark or Tecan Infinite 200 Pro, Tecan Group Ltd., Zürich, Switzerland), background control is the medium with corresponding concentration of glab, licoA, or glycy, and high control is the maximum releasable LDH in enteroids or Caco-2 cells. For enteroids, each well was used as its own positive control by taking the total releasable LDH of each well after incubation.

#### 2.6.2 Cell Viability Assessed by the WST-1 Assay

Effects on cell viability after stimulation by glab, licoA, and glycy were assessed by measuring cleavage of the tetrazolium salt WST-1 to formazan catalyzed by cellular mitochondrial dehydrogenases and analyzed using a WST-1 cell viability kit (PromoKine, Heidelberg, Germany), according to the manufacturer’s instructions. Cell viability was expressed as the percentage of the control cells (enteroids or Caco-2 cells grown in a medium) and was calculated with eq 2.
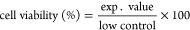
2in which exp. value is the UV absorbance at 450 nm (Tecan Spark or Tecan Infinite 200 Pro) and low control is the spontaneous cleavage of WST-1 to formazan by mitochondrial dehydrogenases in untreated enteroids or Caco-2 cells.

For apical-in enteroids, the cleavage of WST-1 to formazan was assessed in the MG. For this, formazan was released from MG by incubation with GCDR on ice for 10 min, after which the enteroid suspension was centrifuged (250*g*, 3 min, 4 °C), and the supernatant was measured spectrophotometrically at 450 nm (eq 2).

### 2.7 Evaluation of Epithelial Barrier Integrity in Apical-Out Enteroids after Exposure to Glabridin, Licochalcone A, and Glycyoumarin

Effects on barrier integrity of apical-out enteroids after glab, licoA, and glycy exposure were evaluated with a dextran diffusion assay, adapted from Co et al.^14^ In brief, apical-out enteroids were incubated for 4 h with 12.5 μg mL^–1^ glab (39 μM), 12.5 μg mL^–1^ licoA (37 μM), and 12.5 μg mL^–1^ glycy (34 μM) (concentrations equivalent to their reported MIC values against Gram-positive bacteria^17^), after which the experimental agent was removed and enteroids were washed with DMEM/F12 and resuspended in a solution of 4 kDa fluorescein isothiocyanate (2 mg mL^–1^, FITC-dextran 4 kDa). Enteroids were allowed to settle by gravity (5 min) into a pellet, the FITC-dextran 4 kDa was aspirated, and enteroids were washed with DMEM/F12 (3 min, 250*g*, RT). Enteroids were suspended in fresh DMEM/F12, and they were imaged with an EVOS FL Auto 2 cell imaging system. Images were analyzed using ImageJ software (version 1.52). Negative and positive controls were apical-out enteroids exposed to the medium (ODM) or to 2 mM EDTA in PBS (incubated for 15 min on ice), respectively, as it was shown that EDTA disrupts tight junctions and results in compromised barrier integrity without cell death.^14^

### 2.8 Biotransformation of Glabridin, Licochalcone A, and Glycycoumarin in Apical-Out and Apical-In Ileal Enteroids

Biotransformation of glab, licoA, and glycy in apical-out and apical-in enteroids was evaluated after 0, 4, and 24 h of incubation. For this, apical-out enteroids were stimulated with 50 μg mL^–1^ (154 μM) glab, or with 100 μg mL^–1^ licoA (296 μM), or 100 μg mL^–1^ glycy (272 μM), and apical-in enteroids were stimulated with 50 μg mL^–1^ glab (154 μM), 50 μg mL^–1^ licoA (148 μM), or 50 μg mL^–1^ glycy (136 μM). At these concentrations, the compounds did not induce cytotoxicity. For apical-out enteroids, parent compounds and their transformation products were assessed on the enteroids’ apical (facing the medium) and basolateral side (inside the enteroids) and intracellularly. In apical-in enteroids, parent compounds and metabolites were assessed on the basolateral side (facing the medium). For the apical release, apical-out enteroids were pelleted (250*g*, 3 min, 4 °C), and the supernatant was used for LC-MS analysis, after which the enteroids were washed with PBS. Enteroids in a suspension in fresh PBS were broken up by vigorously pipetting up and down approximately for 20 times, after which enteroids were pelleted and the supernatant was used to determine basolateral release by using LC-MS. Enteroids were washed with PBS, and the enteroids’ cells in fresh PBS were disrupted on ice with a digital sonifier (Branson Ultrasonics Corporation, Danbury, CT, USA) with the following settings: a 5 s pulse, a 10 s pause, an amplitude of 55%, and 12 cycles. Enteroids were pelleted, and the supernatant was used as a measure for intracellular release and measured with LC-MS.

#### 2.8.1 Reversed-Phase Liquid Chromatography Photodiode Array (RP-UHPLC-PDA)

Samples were separated on a Thermo Vanquish UHPLC system (Thermo Scientific, San Jose, CA, USA) equipped with a pump, a degasser, an autosampler, and a PDA detector, as described elsewhere.^15^ Eluents used were (A) LC-MS-grade MQ + 0.1% FA (v/v) and (B) LC-MS-grade ACN + 0.1% FA (v/v). The elution program was started by running isocratically at 1% B for 1.09 min followed by a 1.09–26.16 min linear gradient to 70% B, a 26.16–27.25 min linear gradient to 100% B, and 27.25–32.70 min isocratically at 100% B. The eluent was adjusted to start conditions in 1.09 min followed by equilibration of 5.45 min.

#### 2.8.2 Electrospray Ionization Ion Trap Mass Spectrometry (ESI-IT-MS^n^)

Mass spectrometric data were acquired using an LTQ Velos Pro linear ion trap mass spectrometer (Thermo Scientific), equipped with a heated ESI probe coupled in-line to the Vanquish UHPLC system, as described elsewhere.^15^ Data were processed using Xcalibur 4.1 (Thermo Scientific).

#### 2.8.3 Quantification of Glabridin, Licochalcone A, Glycycoumarin, and Biotransformation Products

Quantification of glab, licoA, glycy, and produced metabolites was based on UV absorbance at 280 nm (glab), 340–390 nm (licoA), and 320–370 nm (glycy). For this, a seven-point calibration (0.01–150 μg mL^–1^) curve based on external standards of glab, licoA, and glycy (*R*^2^ > 0.999) was used. UV peaks were integrated using the AVALON integration algorithm with the autocalc function (Xcalibur 4.1). Metabolites were quantified as glab, licoA, or glycy equivalents.

### 2.9 Statistical Analysis

Assessment of cytotoxicity (LDH) and cell viability (WST-1), along with differences in biotransformation products after exposure to glab, licoA, and glycy in apical-out and apical-in enteroids, was done using analysis of variance (ANOVA) with GraphPad Prism 9.3.1. (Boston, MA, USA). Normality and equal variances were confirmed by examination of QQ plots and residual plots, respectively. For LDH and WST-1 data, significant differences (*p* < 0.05) were compared to the negative control, without applying multiple comparisons corrections, using Fisher’s LSD. For differences between 4 and 24 h exposure to glab, licoA, and glycy in apical-out and apical-in enteroids, Tukey’s multiple comparisons test was used with a significance threshold set at *p* = 0.05.

## 3 Results

### 3.1 Glabridin, but Not Licochalcone A and Glycycoumarin, Shows a Dose-Dependent Increase in Cytotoxicity and a Decrease in Cell Viability in Human Apical-Out Ileal Enteroids

**Figure 3:**
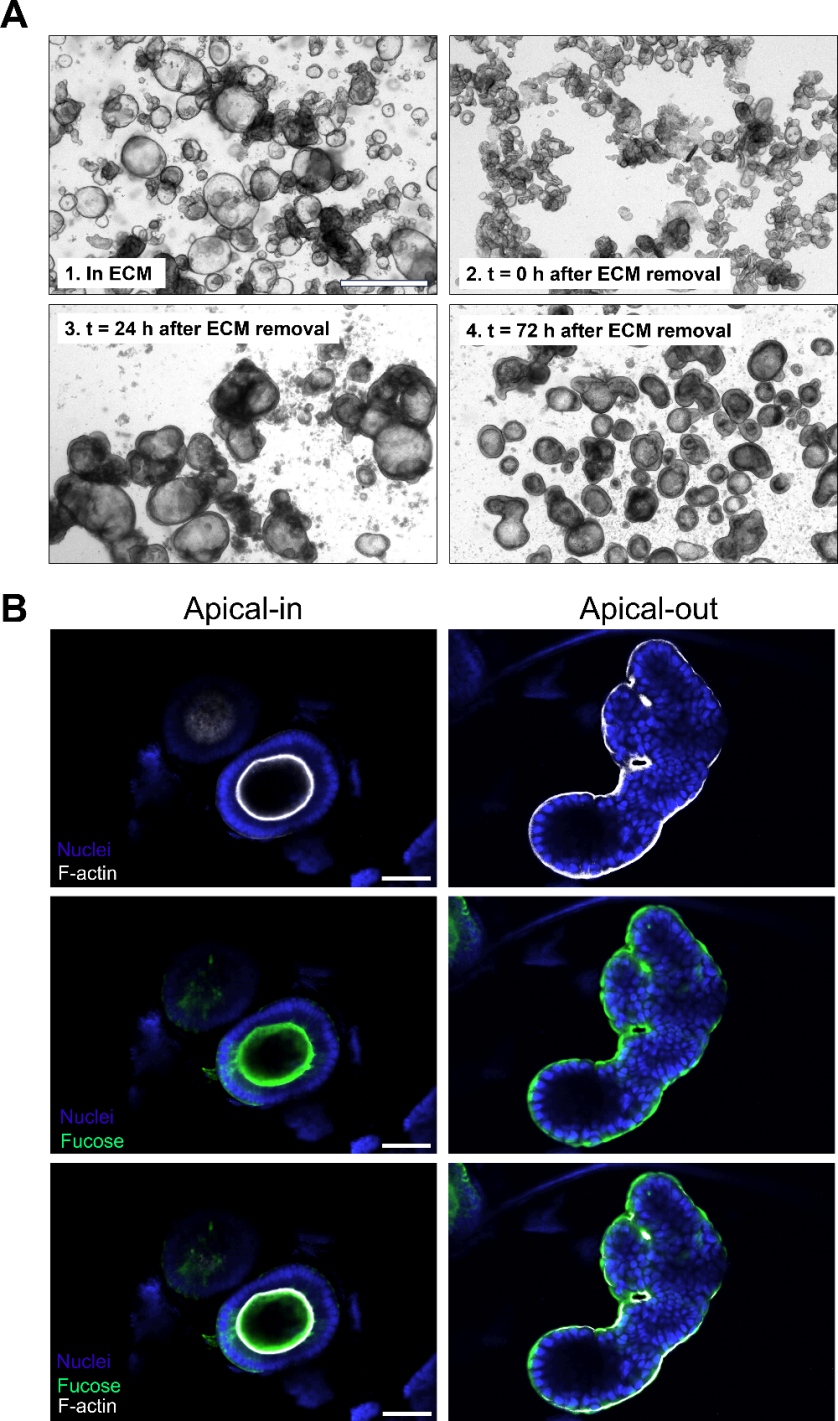
Polarity reversal in human ileal enteroids shown by microscopy imaging. Panel (A) shows brightfield microscopy (4× magnification) of enteroid polarity reversal over time from apical-in to apical-out. Scale bar = 650 μm and applies to all images. Panel (B) shows confocal microscopy (40× magnification) of apical-in (left) and apical-out (right) enteroids. Nuclei were visualized with DAPI (blue), the actin cytoskeleton in the microvilli brush border with Alexa Fluor 660 phallaoidin (white), and fucose units in the mucus layer with *Ulex europaeus* agglutinin I rhodamine (UAE-1) (green). ECM = extracellular protein matrix. Scale bars are 30 μm and apply to all images.

Apical-out ileal enteroids were generated from conventional apical-in enteroids (Figure 3A). Polarity was reversed by removing the ECM, which is known to disrupt interactions between ECM-proteins and basolateral β1-integrin receptors in the enteroids. This triggered a coordinated movement of the epithelium and resulted in eversion of enteroid polarity without alterations to individual cells.^13,14^ Polarity reversal was followed over time in which enteroids fully reversed their polarity 72 h after ECM removal (Figure 3A.4). Changing enteroid polarity from apical-in to apical-out was confirmed by staining F-actin in the apical microvilli brush border. The microvilli brush border moved from the inside of the apical-in enteroid (Figure 3B, left pictures, white layer) toward the outside of the apical-out enteroid (Figure 3B, right pictures, white layer). Additionally, as we previously showed for mouse jejunal apical-out enteroids, human ileal apical-out enteroids exhibit a fucose-containing mucus layer around the apical brush border facing the outside of the enteroids (Figure 3B, green layer).^15^ In apical-in enteroids, this fucose-containing mucus layer surrounds the lumen at the apical brush border on the inside of the enteroids.

**Figure 4:**
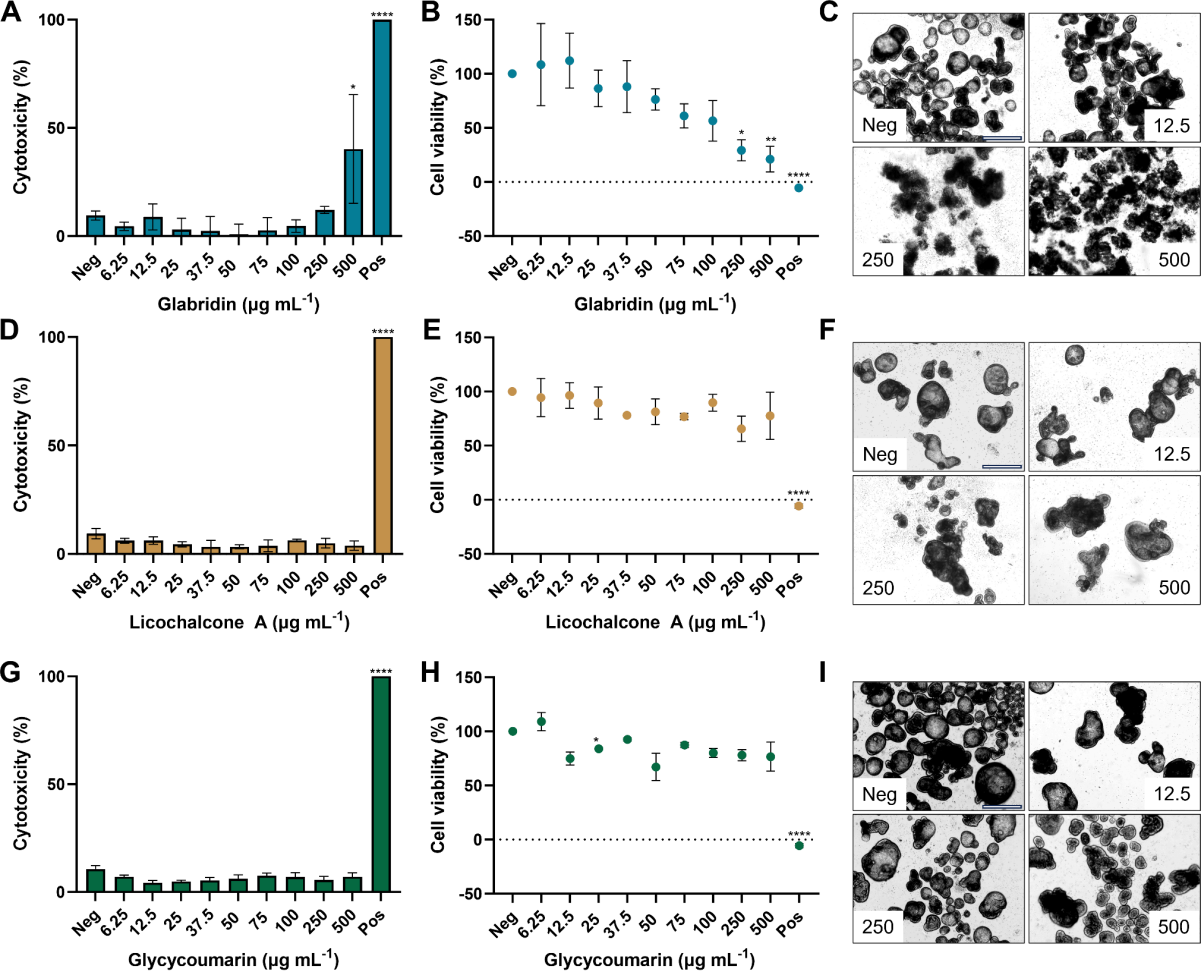
Effects on cytotoxicity and cell viability in human ileal apical-out enteroids after 4 h of glabridin (glab), licochalcone A (licoA), and glycycoumarin (glycy) exposure. Panels (A), (D), and (G) show cytotoxicity (measured by LDH) and panels (B), (E), and (H) show cell viability (measured by WST-1) in μg mL^–1^ after 4 h of exposure to glab, licoA, and glycy, respectively. Brightfield microscopy pictures of apical-out enteroids after 4 h exposure to glab (C), licoA (F), and glycy (I) at various concentrations in μg mL^–1^ are shown, together with the negative control (e.g., apical-out enteroids in ODM). Scale bars are 650 μm and apply to all images. For statistical analysis, data are compared to the negative control (neg) and expressed as the mean ± SEM of at least three biological replicates, measured in duplicate. *p < 0.05, **p < 0.01, ***p < 0.001, and ****p < 0.0001. Neg = negative control and pos = positive control (apical-out enteroids treated with 1% Triton X-100). Microscopy pictures are at a 4× magnification. Additional microscopy pictures after glab, licoA, and glycy exposure are shown in Figures S1, S2, and S3, Supporting Information, respectively. A representative example of cell counts of apical-out ileal enteroids after glycy exposure is shown in Figure S4, Supporting Information.

Next, effects on cytotoxicity and cell viability after 4 h of exposure of a concentration range of 6.25 up to 500 μg mL^–1^ of glab, licoA, or glycy were assessed in apical-out enteroids (Figure 4). No significant effects on cytotoxicity were found for licoA and glycy up to the highest concentrations (*p* > 0.05) (Figure 4D,G). However, for glab, we observed significant cytotoxicity effects at the highest tested concentration, with 31% cytotoxicity at 500 μg mL^–1^ (1541 μM) (compared to the negative control). In addition, effects on cell viability (mitochondrial activity, as measured by WST-1) after 4 h of exposure were determined: licoA (Figure 4E) showed a slight decrease in cell viability to approximately 80%, albeit not statistically significant compared to the negative control (set at 100% viability). Glycy (Figure 4H) followed a similar trend; however, a significant reduction in cell viability was observed at 25 μg mL^–1^ (68 μM). Nonetheless, higher concentrations did not show statistically significant differences, suggesting that the observed trend may be attributed to experimental variability rather than a biological effect. Conversely, glab demonstrated a clear dose–response relationship between the concentration and cell viability (Figure 4B). Up to 100 μg mL^–1^ (308 μM), a decreased trend in cell viability was observed, whereas exposure at 250 μg mL^–1^ (771 μM) and 500 μg mL^–1^ (1541 μM) glab significantly decreased enteroid cell viability to 29 and 21% (*p* < 0.05), respectively. Brightfield microscopy pictures were consistent with our findings on cell viability, but less with the observed cytotoxicities (Figure 4 and Figure S1 for glab, Figure S2 for licoA, and Figure S3 for glycy in the Supporting Information). Specifically, with glab exposure, apical-out enteroids visually showed reduced enteroid integrity and dead cells at 250 μg mL^–1^ (771 μM) and 500 μg mL^–1^ (1541 μM) (Figure 4C and Figure S1, Supporting Information). It should be noted, however, that apical-out enteroids shed dead intestinal cells from the villus tip to the intestinal lumen into the enteroid medium (the human small intestinal epithelium turnover takes about 4–5 days^23^).

### 3.2 Glabridin, Licochalcone A, and Glycycoumarin Show a Dose-Dependent Increase in Cytotoxicity and a Decrease in Cell Viability in Human Apical-In Ileal Enteroids

**Figure 5:**
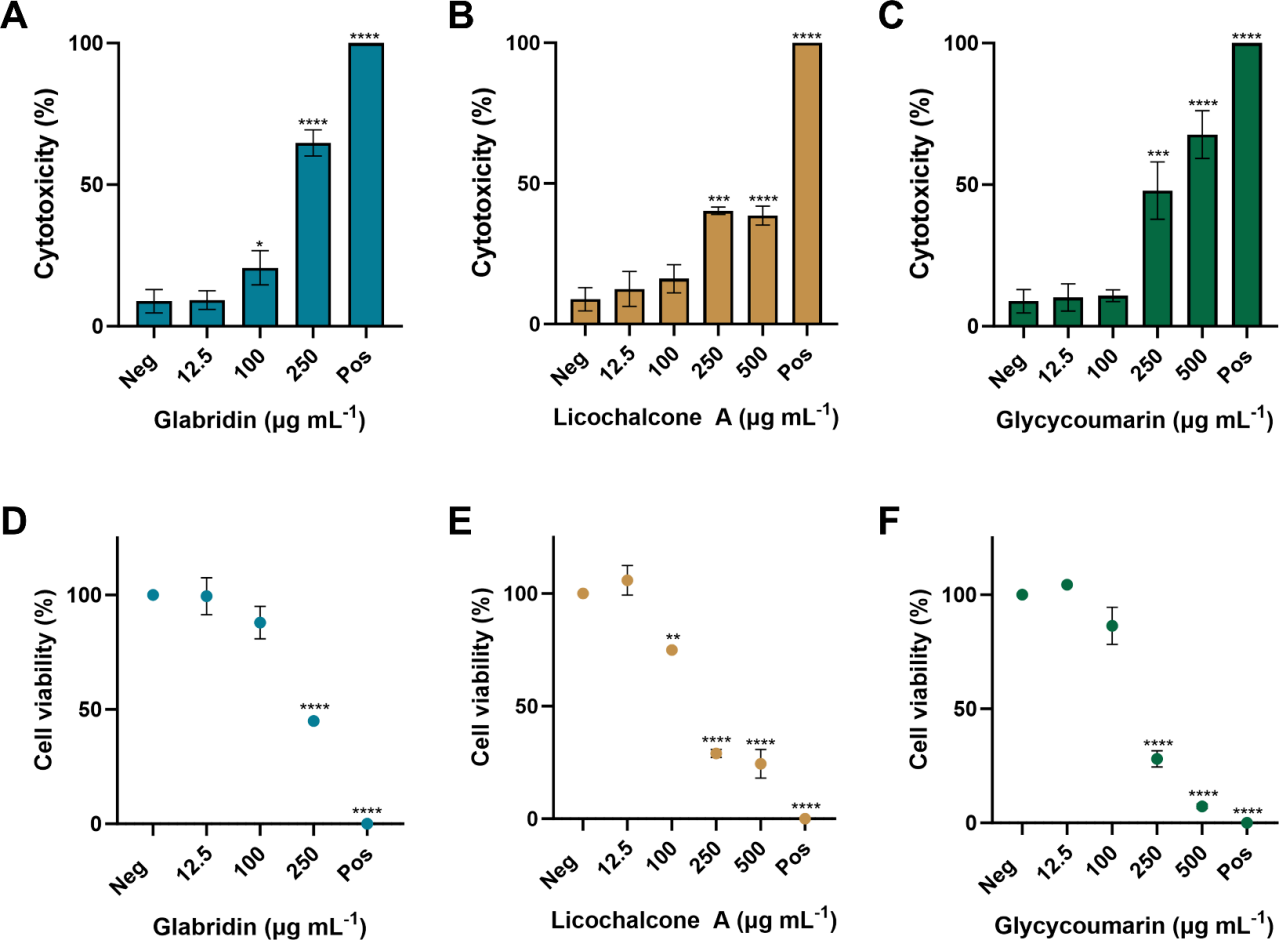
Effects on markers for cytotoxicity and cell viability in human ileal apical-in enteroids after 4 h of glabridin (glab), licochalcone A (licoA), and glycycoumarin (glycy) exposure. Panels (A), (B), and (C) show cytotoxicity (measured by LDH) and panels (D), (E), and (F) show cell viability (measured by WST-1) in μg mL^–1^ after 4 h of exposure to glab, licoA, and glycy, respectively. For the statistical analysis, data are compared to the negative control (neg) and expressed as the mean ± SEM of three biological replicates, measured in duplicate. *p < 0.05, **p < 0.01, ***p < 0.001, and ****p < 0.0001. Neg = negative control (apical-in enteroids in the ODM) and pos = positive control (apical-in enteroids treated with 1% Triton X-100). Brightfield microscopy pictures after glab, licoA, and glycy exposure are shown in Figure S5, Supporting Information.

We compared the cytotoxicity and effects on cell viability after 4 h of exposure to glab, licoA, and glycy in apical-out enteroids with those observed in apical-in enteroids (Figure 5). Microscopy pictures of apical-in enteroids after glab, licoA, and glycy exposure are shown in Figure S5, Supporting Information.

Exposure to 100 μg mL^–1^ glab (308 μM, Figure 5A), 250 μg mL^–1^ licoA (739 μM, Figure 5B), and 250 μg mL^–1^ glycy (679 μM, Figure 5C) significantly increased levels of the cytotoxicity marker in apical-in enteroids compared to the negative control (apical-in enteroids grown in ODM). Enteroid cell viability was significantly decreased at 250 μg mL^–1^ after glab exposure (771 μM, Figure 5D), 100 μg mL^–1^ after licoA exposure (296 μM, Figure 5E), and 250 μg mL^–1^ after glycy exposure (679 μM, Figure 5F).

### 3.3 Exposure to Glabridin, Licochalcone A, and Glycycoumarin at Minimum Inhibitory Antibacterial Concentrations Does Not Impair Epithelial Barrier Integrity in Human Apical-Out Ileal Enteroids

**Figure 6:**
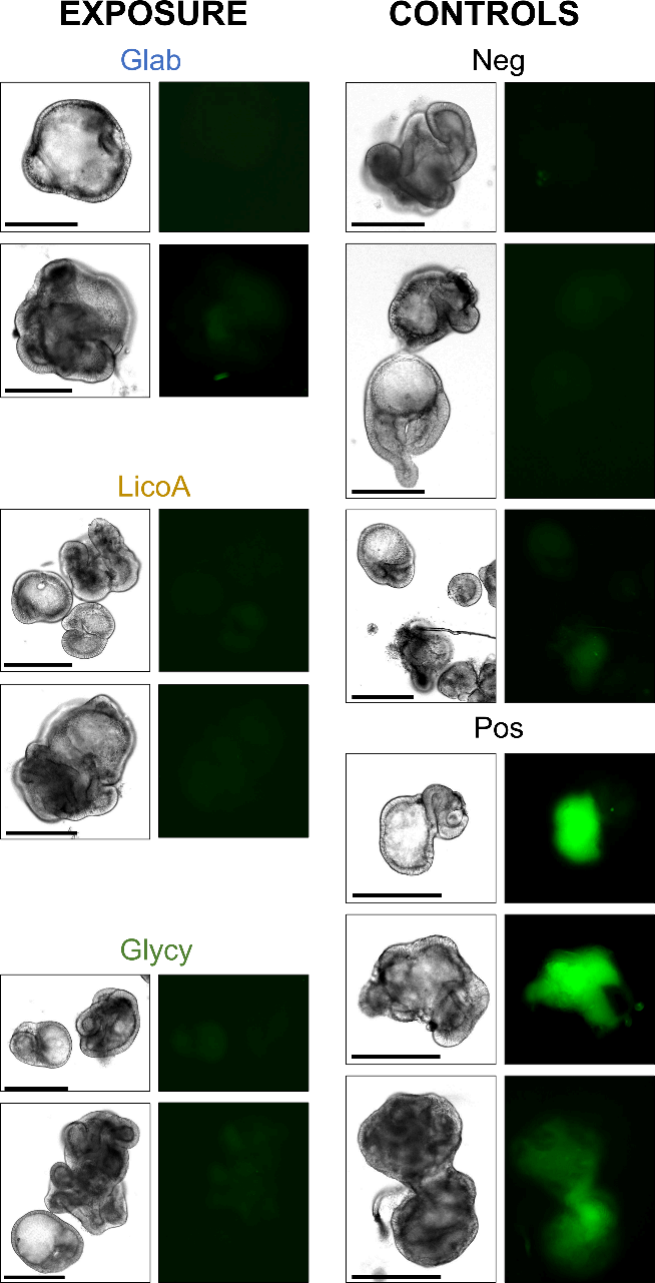
Effects on ileal apical-out barrier integrity after exposure to glabridin (glab), licochalcone A, and glycycoumarin (glycy). The left panels of each pair show brightfield microscopy pictures at a 10× magnification of representative examples of ileal apical-out enteroids exposed to 12.5 μg mL^–1^ glab, licoA, or glycy, negative control (neg, apical-out enteroids in ODM), or positive control (pos, apical-out enteroids in 2 mM EDTA) exposed to a FITC-dextran diffusion assay. Right images show the corresponding barrier integrity images obtained by visualization of FITC-dextran. Scale bars are 275 μm. Experiments were performed in three biological replicates.

To determine the integrity of the enteroid epithelial barrier after 4 h of exposure to glab, licoA, and glycy at their reported antibacterial minimum inhibitory concentrations,^17^ we performed a FITC-dextran (of 4 kDa) diffusion assay (Figure 6).

Apical-out enteroids that were not exposed to prenylated phenolics (negative control: Neg, Figure 6) excluded FITC-dextran, indicating that the tight junctions formed a tight seal, preventing passage of FITC-dextran through the epithelial monolayer. In contrast, barrier integrity in apical-out enteroids was disrupted after exposure to the chelating agent EDTA (positive control: Pos, Figure 6). FITC-dextran diffused into the intercellular spaces and into the center of the enteroids and subsequently resulted in bright fluorescent enteroids. Exposure of the ileal apical-out enteroids to glab, licoA, and glycy at their reported antibacterial minimum inhibitory concentrations (12.5 μg mL^–1^ or 34–39 μM)^17^ did not seem to disrupt the enteroids’ membrane integrity, as the enteroids were visually similar to the negative control.

### 3.4 Human Apical-Out Ileal Enteroids Show Apical Release of Phase II Biotransformation Products after Exposure to Glabridin, Licochalcone A, and Glycycoumarin

**Figure 7:**
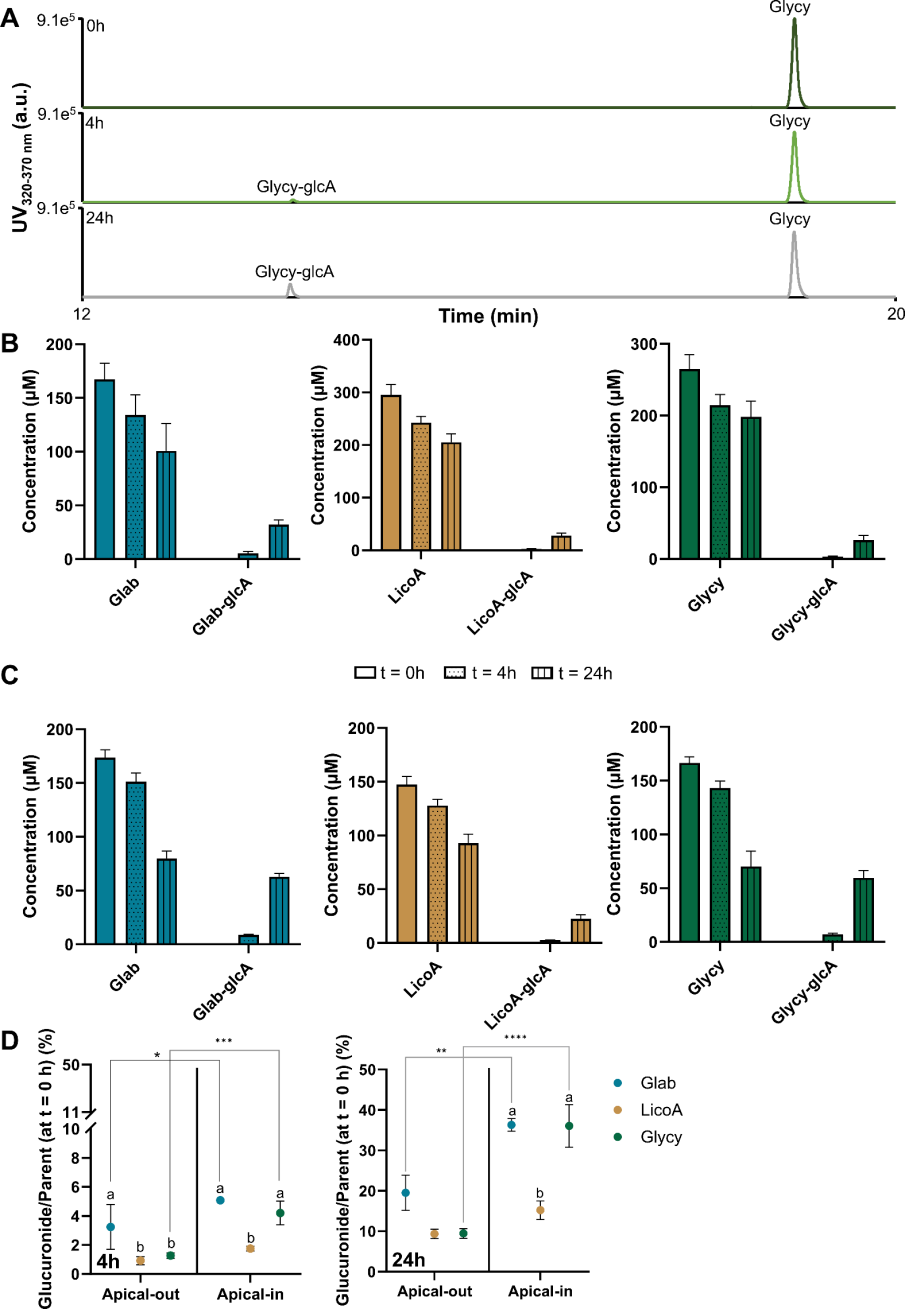
Biotransformation of glabridin (glab), licochalcone A (licoA), and glycycoumarin (glycy) in human apical-out and apical-in ileal enteroids. (A) Representative UV chromatograms (320–370 nm) between 12 and 20 min of the biotransformation of glycy in apical-out enteroids at *t* = 0 h (top), *t* = 4 h (middle), and *t* = 24 h (bottom) between 12 and 20 min, (B) biotransformation of glab (50 μg mL^–1^ or 154 μM), licoA (100 μg mL^–1^ or 296 μM), and glycy (100 μg mL^–1^ or 271 μM) in apical-out enteroids at *t* = 0 h, *t* = 4 h, and *t* = 24 h measured at the apical side, (C) biotransformation of glab (50 μg mL^–1^ or 154 μM), licoA (50 μg mL^–1^ or 148 μM), and glycy (50 μg mL^–1^ or 136 μM) in apical-in enteroids at *t* = 0 h, *t* = 4 h, and *t* = 24 h measured at the basolateral side, and (D) percentage of glucuronide biotransformation products after 4 h (left panel) and 24 h (right panel) compared to the untransformed parent at *t* = 0 h. % Biotransformation is calculated as the ratio between the metabolite and the parent compound at *t* = 0 h in μM. Glab-glcA = glabridin-glucuronide, licoA-glcA = licochalcone A-glucuronide, and glycy-glcA = glycycoumarin-glucuronide. Data are expressed as the mean ± SEM of at least three biological replicates measured in duplicate. For statistical analyses in panel (D), letters indicate significant differences (*p* < 0.05) within the same model, and stars indicate significant differences (*p* < 0.05) between models (apical-out vs apical-in enteroids). *p < 0.05, **p < 0.01, ***p < 0.001, and ****p < 0.0001.

In addition to cytotoxicity effects, cell viability, and barrier function, we determined the biotransformation of glab, licoA, and glycy in apical-out enteroids and compared these with apical-in enteroids (Figure 7). In apical-out enteroids, the parent compounds and biotransformation products were measured on the apical and basolateral sides as well as intracellularly. Figure 7B shows the biotransformation products (after exposure to nontoxic concentrations of glab (50 μg mL^–1^ or 154 μM), licoA (100 μg mL^–1^ or 296 μM), and glycy (100 μg mL^–1^ or 271 μM)) that were released on the apical side of apical-out enteroids at 0, 4, and 24 h of exposure to glab, licoA, and glycy. The release of biotransformation products at the basolateral side in apical-in enteroids after exposure to nontoxic concentrations of glab (50 μg mL^–1^ or 154 μM), licoA (50 μg mL^–1^ or 148 μM), and glycy (50 μg mL^–1^ or 136 μM) is shown in Figure 7C. All biotransformation products were identified with RP-UHPLC-PDA-ESI-IT-MS^n^, and annotations are shown in Table S1 (Supporting Information).

Apical-out ileal enteroids metabolized glab, licoA, and glycy into their glucuronide metabolites, which were released on the enteroids’ apical side. Basolateral release and intracellular concentrations of glucuronides were negligible with <0.1 μM after 24 h of incubation. Glab, licoA, and glycy were stable in the medium (without enteroids), and no spontaneous metabolism was observed, as was confirmed with RP-UHPLC-PDA-MS^n^ analysis. Glab, licoA, and glycy showed recoveries of 101 ± 2.2, 101 ± 3.2, and 101 ± 3.0%, respectively, after 24 h of incubation.

Apical-out ileal enteroids transformed glycy (265 μM at *t* = 0 h) to glycy-glucuronide after 4 h (∼3 μM) and 24 h (∼16 μM) (Figure 7A,B). No phase I biotransformation products (e.g., hydroxylation) were observed in apical-out enteroids up to 24 h of glab, licoA, and glycy incubation. Apical-in enteroids showed similar processes, where glab, licoA, and glycy were transformed to their glucuronides (Figure 7C), and no phase I metabolites were identified. Here, biotransformation products were identified at the basolateral side of the enteroids.

After 24 h of exposure to glab and glycy, apical-out enteroids released significantly less biotransformation products to their surrounding environment compared to apical-in enteroids (*p* < 0.05) (Figure 7D, right panel). For example, glycy showed extensive biotransformation in apical-in enteroids with transformation of approximately 40% compared to glycy at *t* = 0 h, whereas biotransformation in apical-out enteroids was ∼10% (*p* < 0.0001). Biotransformation after 24 h of exposure to licoA was comparably low in both enteroid models, with no significant differences observed (*p* > 0.05).

### 3.5 Human Apical-Out Enteroids Are More Resilient toward Exposure to Prenylated Phenolics than Apical-In Enteroids and Caco-2 Cells

**Figure 8:**
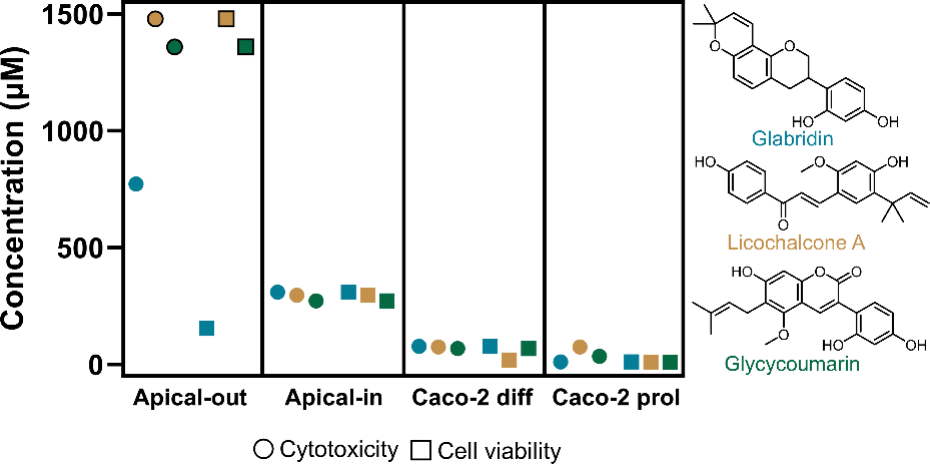
Comparisons of cytotoxicity and cell viability of glabridin (glab, blue), licochalcone A (licoA, yellow), and glycycoumarin (glycy, green) in different *in vitro* intestinal systems. Schematic summary of the highest nontoxic concentrations (circles, measured with LDH) and nonreducing cell viabilities (squares, measured with WST-1) in human apical-out enteroids, human apical-in enteroids, differentiated Caco-2 cells (Caco-2 diff),^17^ and proliferating Caco-2 cells (Caco-2 prol, Figure S6, Supporting Information). A cutoff threshold of 25% cytotoxicity or reduced viability compared to the negative control was used. Circles with a black outline are the highest tested concentrations that did not show effects (>25%) on cytotoxicity and cell viability (Figure 4). Direct comparisons in cell viability after glab, licoA, and glycy exposure in apical-out and apical-in enteroids are shown in Figure S7, Supporting Information.

Lastly, we compared the effects on cytotoxicity and cell viability after exposure to glab, licoA, and glycy in different *in vitro* systems, including human apical-out enteroids (Figure 4), human apical-in enteroids (Figure 5), proliferating Caco-2 cells (representative of the colon) (Figure S6, Supporting Information), and differentiated Caco-2 cells (representative of the small intestine).^17^ A summary is given in Figure 8 comparing the highest non-cytotoxic concentrations and highest concentrations where cell viability was not reduced for the different models. This determination was based on a cutoff value of 25%, relative to the negative control set at 100%, and hence, it may not necessarily represent the initial significant cytotoxicity or significant reduced cell viability value. To further demonstrate the differences in cell viability between apical-out and apical-in enteroids after exposure to various concentrations of glab, licoA, and glycy, we refer to Figure S7 (Supporting Information).

In Figure 8, the cytotoxicity assessment (LDH) revealed that apical-out enteroids were more resilient to exposure to glab, licoA, and glycy compared to apical-in enteroids. The highest nontoxic concentrations were >1500 μM for licoA and glycy (500 μg mL^–1^) and 771 μM (250 μg mL^–1^) for glab in apical-out enteroids, while they were around 300 μM in apical-in enteroids.

Analyzing the effects on cell viability (WST-1), apical-out enteroids displayed a greater resistance to licoA and glycy, with the highest nonreducing viability concentrations being ≥1478 μM (500 μg mL^–1^) and 1357 μM (500 μg mL^–1^), respectively. Conversely, apical-out enteroids were more susceptible to glab exposure, with the highest nonreducing viability observed at 154 μM (50 μg mL^–1^). Notably, at concentrations higher than 100 μg mL^–1^, differences between the models were observed, where cell viability markedly decreased in apical-in enteroids but was less pronounced in apical-out enteroids (Figure S7).

Caco-2 cells (proliferating and differentiated^17^) were proven to be most vulnerable to exposure to glab, licoA, and glycy, with established highest nontoxic concentrations around 75 μM and with even lower nonreducing viability concentrations.

## 4 Discussion

In this study, we elucidated that structurally related prenylated phenolics from licorice roots exhibit differential effects on the intestinal epithelium by using apical-out enteroids, the most physiologically relevant *in vitro* model to date. We demonstrated that human ileal enteroids with an apical-out orientation provide a physiologically relevant (fucose-containing mucus layer surrounding the apical brush border on their exposure side) and suitable model to study cytotoxicity, cell viability, and interactions with the intestinal epithelial layer of bioactive compounds, e.g., those present in food, in this case prenylated phenolics from licorice roots. Furthermore, we have shown that apical-out enteroids possess specialized intestinal functionalities, including barrier function and biotransformation capacity, which is consistent with previous research.^13,14,24^ Apical-out enteroids are advantageous over apical-in enteroids in terms of easy accessibility to the apical or luminal surface and no diffusion restrictions in the ECM scaffold.^13,14,24,25^ Additionally, the orientation of the mucus layer toward the side of exposure increases the models’ representation of the *in vivo* situation in terms of compound–epithelial interactions. Together with our recent findings in mouse jejunal enteroids,^15^ where we showed that apical-out enteroids represent the *in vivo* small intestine better^16^ than apical-in enteroids (based on the gene expression of epithelial cell markers), we believe that apical-out human enteroids are superior in terms of recapitulating the *in vivo* situation. Thus, using apical-out enteroids offers a more accurate alternative to traditional *in vitro* models.

### 4.1 The Intestinal Epithelium Is More Sensitive toward Glabridin Compared to Licochalcone A and Glycycoumarin

Apical-out enteroids were less susceptible to exposure to glab, licoA, and glycy compared to apical-in enteroids, with significant effects on cytotoxicity found at 3–5-fold higher concentrations in the apical-out model. We hypothesize that the hydrophilic mucus layer surrounding the apical-out enteroids hinders the diffusion of hydrophobic glab, licoA, and glycy, limiting their exposure to the epithelial cells. In apical-in enteroids, glab, licoA, and glycy do not encounter the mucus layer on the exposure side since the mucus layer is located on the inside of the enteroid, and thus, compounds will reach the epithelial cells more easily. In terms of cell viability, apical-out enteroids were more resilient toward licoA and glycy exposure than apical-in enteroids. This difference between models was not observed for glab, wherein comparable susceptibilities between models were observed. Glab impacted the mitochondrial activity of epithelial cells at lower concentrations compared with licoA and glycy, which suggests the potential induction of cellular stress that is often associated with impaired energy metabolism and cellular function.

Of the three tested compounds that we assessed in this study, we observed that apical-out enteroids showed an increased susceptibility toward glab followed by licoA and glycy. Apical-out enteroids were exposed to the compounds at physiological pH (pH ∼7.4). At this pH, the compounds exist in an equilibrium of dissociated (negatively charged) and undissociated (neutral) forms, depending on the p*K*_a_ values of the hydroxyl groups on the phenolic backbone (Figure S8, Supporting Information). At physiological pH, glab is present in the undissociated form (>99%), while licoA and glycy are ∼30% and ∼70% dissociated, respectively. We postulate that glab (neutral or uncharged) can better diffuse through the neutral protein regions of the overall negatively charged mucus layer (due to the prevalence of sialic acids and sulfates)^26−28^ and reach the intestinal epithelium more easily compared to licoA and glycy (negatively charged), leading to a higher susceptibility toward glab. In contrast, negatively charged licoA and glycyl groups are likely to be repelled by the mucus layer.

The cytotoxic mode of action (in cell lines) of prenylated phenolics has been linked to intracellular targets, mediating changes in the cell cycle signaling machinery that ultimately lead to reduced cell proliferation and apoptotic cell death.^29−33^ It should be noted, however, that cytotoxicity of prenylated phenolics in cell lines, including in Caco-2 cells in this study,^17,29−35^ was observed at considerably lower concentrations than those observed in apical-out and apical-in enteroids (with the highest nontoxic concentrations around 75 μM in Caco-2 cells compared to generally >1500 μM in apical-out and approximately 300 μM in apical-in enteroids). It is conceivable that these observed differences between cell lines and apical-out enteroids are due to the cell type (and energy metabolism; cancerous vs healthy), the absence of a proper mucus layer, and the lack of specific biotransformation enzymes and transporters.^11,36^

We compared the determined cytotoxicities of glab, licoA, and glycy in both human enteroid models with their minimum inhibitory concentrations (MICs) against a variety of Gram-positive bacteria (including *L. buchneri*, *Streptococcus mutans*, and *S. aureus*), as we have published previously.^17^ For licoA and glycy, the highest nontoxic concentrations were at least 40 times higher than their MIC (against Gram-positive bacteria) in apical-out enteroids. The highest nontoxic concentration after glab exposure was 20-fold higher than the reported MICs. Nevertheless, enteroid cell viability was compromised at lower concentrations, and the concentrations at which cell viability was still unaffected were found to be 3 to 8 times higher than the reported MICs, for glab, licoA, and glycy. While the observed differences between MIC and reduced cell viabilities in apical-out enteroids may appear modest, we did not observe a reduction in barrier integrity after glab, licoA, and glycy exposure at their MIC values. Altogether, these data indicate that there is a window of opportunity for glab, licoA, and glycy as natural antibacterials for food preservation and/or in clinical settings.^19,37^ It should be noted that the exposure time of the enteroids and bacteria toward the prenylated phenolics was different, with 4 and 24 h, respectively. Nevertheless, the average human small intestinal transit time ranges between 2 and 6 h, suggesting that 4 h of incubation is relevant to the *in vivo* situation.^38^ Looking at human *in vivo* data, there is evidence that prenylated phenolics from licorice roots do not show adverse effects at concentrations well above their reported MICs against Gram-positive bacteria. Several studies have shown that daily supplementation with a *G. glabra* extract up to 600 mg per day, equivalent to 18 mg of glab, did not induce adverse effects.^39−41^ Our findings with apical-out enteroids align with the safety outcomes and the subsequent approval of an ethanolic *G. glabra* root extract as a safe novel food ingredient by the EFSA.^41^ Our findings specifically highlight that apical-out enteroids exhibit greater resilience to licoA and glycy than to glab. This suggests that licoA from *G. inflata* and glycy from *G. uralensis* show more promise for future applicability.

### 4.2 Human Enteroids Metabolize Prenylated Phenolics to Phase II Biotransformation Products

Apical-out and apical-in enteroids were able to transform glab, licoA, and glycy to their corresponding glucuronides, a common phase II biotransformation reaction that is catalyzed by UDP-glucuronosyltransferases (UGT). Recently, Kakni and co-workers reported that the gene expression of various UGT enzymes was equally expressed in apical-out and apical-in enteroids.^24^ In this study, we show that both enteroid models can transform prenylated phenolics, indicating that the UGT enzymes in enterocytes exhibit functional activity. Another common phase II biotransformation is sulfation of xenobiotics by sulfotransferases (SULT).^42−44^ Our LC-MS analysis did not reveal sulfation of glab, licoA, and glycy. This finding contrasts with Yokota and co-workers, who identified the expression of several SULT enzymes in (apical-in) duodenal enteroids.^45^ We previously showed that mouse jejunal apical-out and apical-in enteroids were able to sulfate glab (and licoA and glycy in apical-in enteroids), albeit to minor concentrations compared to their glucuronide products.^15^ It is therefore likely that SULT enzymes are present in human ileal enteroids but that the minor concentrations fall below the detection limits of our analytical method.

Besides phase II biotransformation, we specifically searched for phase I biotransformation products, including hydroxy metabolites. Different enzymes play a role during phase I biotransformation, but cytochrome P450 (CYP450) enzymes are key players.^46−48^ We did not identify any phase I biotransformation products in both ileal enteroid models up to 24 h of exposure to glab, licoA, and glycy, which is opposite to what we observed for licoA and glycy in apical-in mouse jejunal enteroids.^15^ The absence of phase I biotransformation is likely explained by the inhibition of human CYP enzymes (i.e., CYP3A4) by glab,^49−51^ licoA,^50,52^ and glycy.^50^ It is worth mentioning that drug-metabolizing enzymes, such as CYP enzymes (i.e., 3A4, 2C9, and 2J2), are present and active in human colon apical-out and apical-in organoids.^24^ Therefore, human enteroids provide a promising model for intestinal biotransformation, as opposed to Caco-2 cells, which have been reported to lack CYP enzymes.^11,36^

Glab, licoA, and glycy glucuronides were mainly present on the apical side of apical-out and on the basolateral side of apical-in enteroids. In apical-out enteroids, we did not identify (or only minor concentrations of) metabolites on the basolateral side. Based on the concentrations of metabolites quantified on the basolateral side in apical-in enteroids, we postulate that no or minor amounts of metabolites would be present on their apical side. This seemingly unexpected result that metabolites are detected on the apical side in apical-out enteroids and on the basolateral side of apical-in enteroids may be explained by variations in the expression of transporters in the intestinal epithelium between both enteroid models. Recent findings suggest that apical-out enteroids show a higher expression of apical transporters and that apical-in enteroids a higher expression of basolateral transporters.^24^ We speculate that glab, licoA, and glycy and metabolites are actively excreted from apical-out and apical-in enteroids by apical transporters (e.g., P-glycoprotein, multidrug resistance proteins [MRPs], and cancer resistance proteins)^49,53−55^ and basolateral transporters (e.g., MRPs), respectively.^54,55^ The more pronounced biotransformation (after 24 h) of glab, licoA, and glycy in apical-in enteroids compared with apical-out enteroids is expected to be caused by the absence of a hydrophilic mucus layer on the exposure side in apical-in enteroids, which does not limit diffusion of hydrophobic prenylated phenolics to the enterocytes.

### 4.3 Enteroid Models Can Meet the Demand for *In Vitro* Gut Models that Closely Mimic the Epithelial Morphology and Intestinal Functionality

We have found that apical-out enteroids (Section 3.5) were more robust toward exposure to prenylated phenolics than Caco-2 cells in terms of cytotoxicity.^17^ Also, apical-in enteroids that lacked a mucus layer on the exposure side were less susceptible toward prenylated phenolics than Caco-2 cells. Apart from inherent differences in the cellular state (cancerous vs healthy), we speculate that cell lines such as Caco-2 cells are more sensitive due to factors such as the absence of a proper mucus layer, a lack of biotransformation enzymes and transporters,^11,36^ and an increased surface area by the presence of microvilli on the apical side.^56^ Therefore, we believe that apical-out enteroids as used here can provide added value when combined with the existing established models like Caco-2, which are primarily valuable for screening and have the advantage of being easier to obtain, set up, and standardize. We show here that OECD toxicity methods (i.e., LDH and WST-1) are effective in apical-out enteroids. Thus, apical-out enteroids might form in the future a basis for a testing model that can be complementary to the existing OECD models for toxicity studies.

### 4.4 Human and Mouse Apical-Out Enteroids Show Similar Cytotoxic Responses and Effects on Cell Viability after Exposure to Glabridin

**Figure 9:**
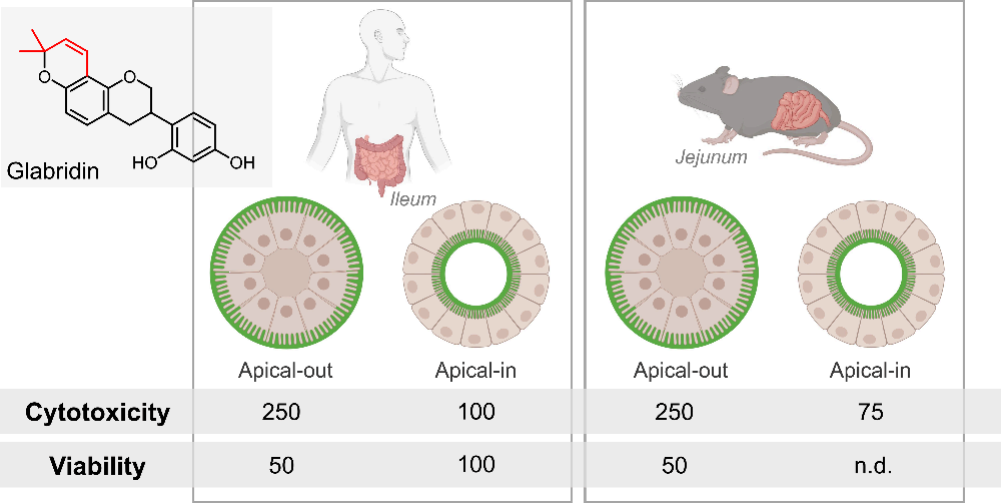
Overview of cytotoxicity and cell viability after exposure to glabridin in human and mouse enteroids. Numbers represent the highest noncytotoxic and nonreducing viability concentrations compared to the negative control (enteroids in a medium) and are reported in μg mL^–1^. A cutoff threshold of 25% cytotoxicity or reduced viability compared to the negative control was used. n.d. = not determined. Figure created with BioRender.com.

We compared all measured cytotoxicity and cell viability data after 4 h of exposure to glab in human apical-out and apical-in ileal enteroids with our previously reported data on cytotoxicity and cell viability after glab exposure in mouse apical-out and apical-in jejunal enteroids.^15^ An overview displaying the highest nontoxic concentrations and highest concentrations where cell viability is not impaired after exposure to glab is given in Figure 9.

Both human and mouse enteroid models showed a comparable response to glab exposure, with apical-out enteroids showing a higher resilience than apical-in enteroids, as illustrated in Figure 9. Therefore, mouse and human apical-out enteroids can probably be considered as suitable models for assessing the cytotoxic effects of prenylated phenolics. Nevertheless, human models outperform mouse models because species differences are circumvented, enhancing the translational relevance of the findings. In this study, this was also evident in the differences in biotransformation between both species.

To conclude, in this study, we demonstrated that human apical-out enteroids, which contain all major gut epithelial cells and proper mucus orientation, provide a physiologically relevant model. The fact that their morphology and functionality more closely resemble the *in vivo* situation apparently results in different values compared to established cell line-based models (i.e., Caco-2) for *in vitro* cytotoxicity and cell viability testing, with the latter suggesting epithelial cytotoxicity at much lower concentrations. Despite and at the same time due to the complexity of our model, it can therefore be of added value in research on compound functionality and *in vitro* cytotoxicity. The response to glab, licoA, and glycy at previously reported MIC values against Gram-positive bacteria did not (i) induce cytotoxicity, (ii) affect cell viability of the enteroids, nor (iii) alter the enteroids’ membrane integrity. Our results using human apical-out enteroids suggest that prenylated phenolics hold promise for various applications, such as in food preservation. Notably, licoA and glycy seemed to be more suitable for potential future applications. Importantly, these compounds exhibit no harmful intestinal interactions, as tested with our model at MIC values where they exhibit antibacterial activity.

## Supporting Information Available

(Figures S1–S3) Brightfield and fluorescence microscopy pictures of apical-out ileal enteroids after glabridin, licochalcone A, and glycycoumarin exposure; (Figure S4) Representative cell counts of apical-out ileal enteroids for cytotoxicity and cell viability experiments; (Table S1) (tentative) identification of glabridin, licochalcone A, and glycycoumarin and biotransformation products in ileal enteroids with RP-UHPLC-PDA-MS^n^; (Figure S5) brightfield microscopy pictures of apical-in ileal enteroids after glabridin, licochalcone A, and glycycoumarin exposure; (Figure S6) cytotoxicity and effects on cell viability of glabridin, licochalcone A, and glycycoumarin on proliferating Caco-2 cells; (Figure S7) comparisons in cell viability between human apical-out and apical-in enteroids after glabridin, licochalcone A, and glycycoumarin exposure; (Figure S8) molecular characteristics of glabridin, licochalcone A, and glycycoumarin; (Table S2) overview of the experimental conditions for exposure experiments with glabridin, licochalcone A, and glycycoumarin in the different cell model (PDF)

## Abbreviations

ACN: acetonitrile
ANOVA: analysis of variance
BSA: bovine serum albumin
Caco-2: human colon adenocarcinoma
CYP: cytochrome P450
DAPI: 4′,6-diamidino-2-phenylindole
DAPT: *N*-[2*S*-(3,5-difluorophenyl)acetyl]-l-alanyl-2-phenyl-1,1-dimethylethylester
diff: differentiated
DMEM: Dulbecco’s modified Eagle’s medium
DMSO: dimethyl sulfoxide
ECM: extracellular protein matrix
EDTA: ethylenediamine tetra acetic acid
FA: formic acid
FCS: fetal calf serum
FITC: fluorescein isothiocyanate
GCDR: gentle cell dissociation reagent
glab: glabridin
GlcA: glucuronic acid
glycy: glycycoumarin
HEPES: 4-(2-hydroxyethyl)-1-piperazineethanesulfonic acid
LDH: lactate dehydrogenase
licoA: licochalcone A
MeOH: methanol
MG: Matrigel
MIC: minimum inhibitory concentration
Neg: negative control
METC: Medical Ethical Review Committee
MRP: multidrug resistance proteins
MQ: Milli-Q water
ODM: organoid differentiation medium
OGM: organoid growth medium
PBS: phosphate-buffered saline
prol: proliferating
pos: positive control
P/S: penicillin/streptomycin
RP-UHPLC-PDA-ESI-IT-MS^n^: reversed-phase ultrahigh-pressure liquid chromatography photodiode array electrospray ionization ion trap mass spectrometry
RT: room temperature
SEM: standard error of the mean
SULT: sulfotransferase
UAE-1: *Ulex europaeus* agglutinin I rhodamine
UGT: UDP-glucuronosyltransferase
WST-1: (4-[3-(4-iodophenyl)-2-(4-nitrophenyl)-2*H*-5-tetrazolio]-1,3-benzenesulfonate)
ZGV: Hospital Gelderse Vallei
