# Supplementary material for: Biotransformation and Epithelial Toxicity of Prenylated Phenolics from Licorice Roots (Glycyrrhiza spp.) in 3D Apical-Out Mucus-Producing Human Enteroids
Source: J Agric Food Chem. 2024 Sep 6;72(37):20396–409. doi: 10.1021/acs.jafc.4c03120 (PMC11421016; doi:10.1021/acs.jafc.4c03120)
Supplement: Supplementary file 1 — jf4c03120_si_001.pdf [file jf4c03120_si_001.pdf]

Supplementary information

Biotransformation and epithelial toxicity of prenylated phenolics from licorice roots (*Glycyrrhiza* spp.) in 3D apical-out mucus producing human enteroids

Sarah van Dinteren, Carla Araya-Cloutier, Shanna Bastiaan-Net, Anouk Boudewijn, Tjarda van Heek, Jean-Paul Vincken, Renger Witkamp, Jocelijn Meijerink

Figure S1. Brightfield and fluorescence microscopy pictures of apical-out ileal enteroids after glabridin exposure

Figure S2. Brightfield and fluorescence microscopy pictures of apical-out ileal enteroids after licochalcone A exposure

Figure S3. Brightfield and fluorescence microscopy pictures of apical-out ileal enteroids after glycycoumarin exposure

Figure S4. Representative cell counts of apical-out ileal enteroids for cytotoxicity and cell viability experiments

Table S1. (Tentative) identification of glabridin, licochalcone A, and glycycoumarin and biotransformation products in ileal enteroids with RP-UHPLC-PDA-MS^n

Figure S5. Brightfield microscopy pictures of apical-in ileal enteroids after glabridin, licochalcone A, and glycycoumarin exposure

Figure S6. Cytotoxicity and effects on cell viability of glabridin, licochalcone A, and glycycoumarin on proliferating Caco-2 cells

Figure S7. Comparisons in cell viability between human apical-out and apical-in enteroids after glabridin, licochalcone A, and glycycoumarin exposure

Figure S8. Molecular characteristics of glabridin, licochalcone A, and glycycoumarin

Table S2. Overview of the experimental conditions for exposure experiments with glabridin, licochalcone A, and glycycoumarin in the different cell model

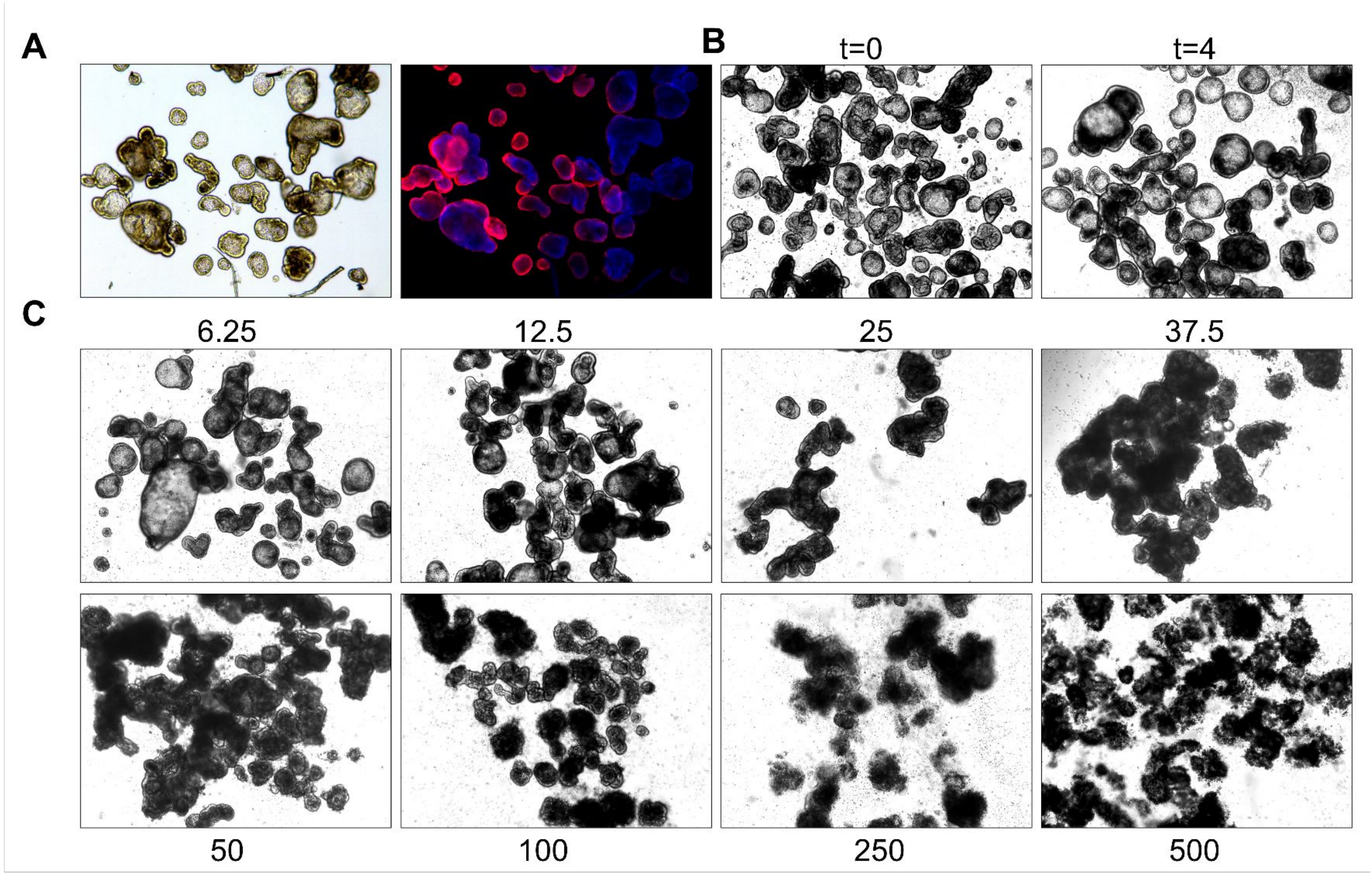

Figure S1. **Microscope pictures of apical-out ileal enteroids after glabridin (glab) exposure**. Panel (A) shows brightfield and fluorescence microscopy pictures of apical-out ileal enteroids, in which nuclei are stained with DAPI (blue) and actin in the microvilli brush border with ActinRed^{TM} 555 (red). (B) shows apical-out enteroids that are not exposed to glab (negative control) at t = 0 h and t = 4 h, and (C) shows apical-out enteroids that are exposed to different glab concentrations. Concentrations are shown above and below pictures and are in μg mL^{-1}. All pictures are shown with 4 x magnification.

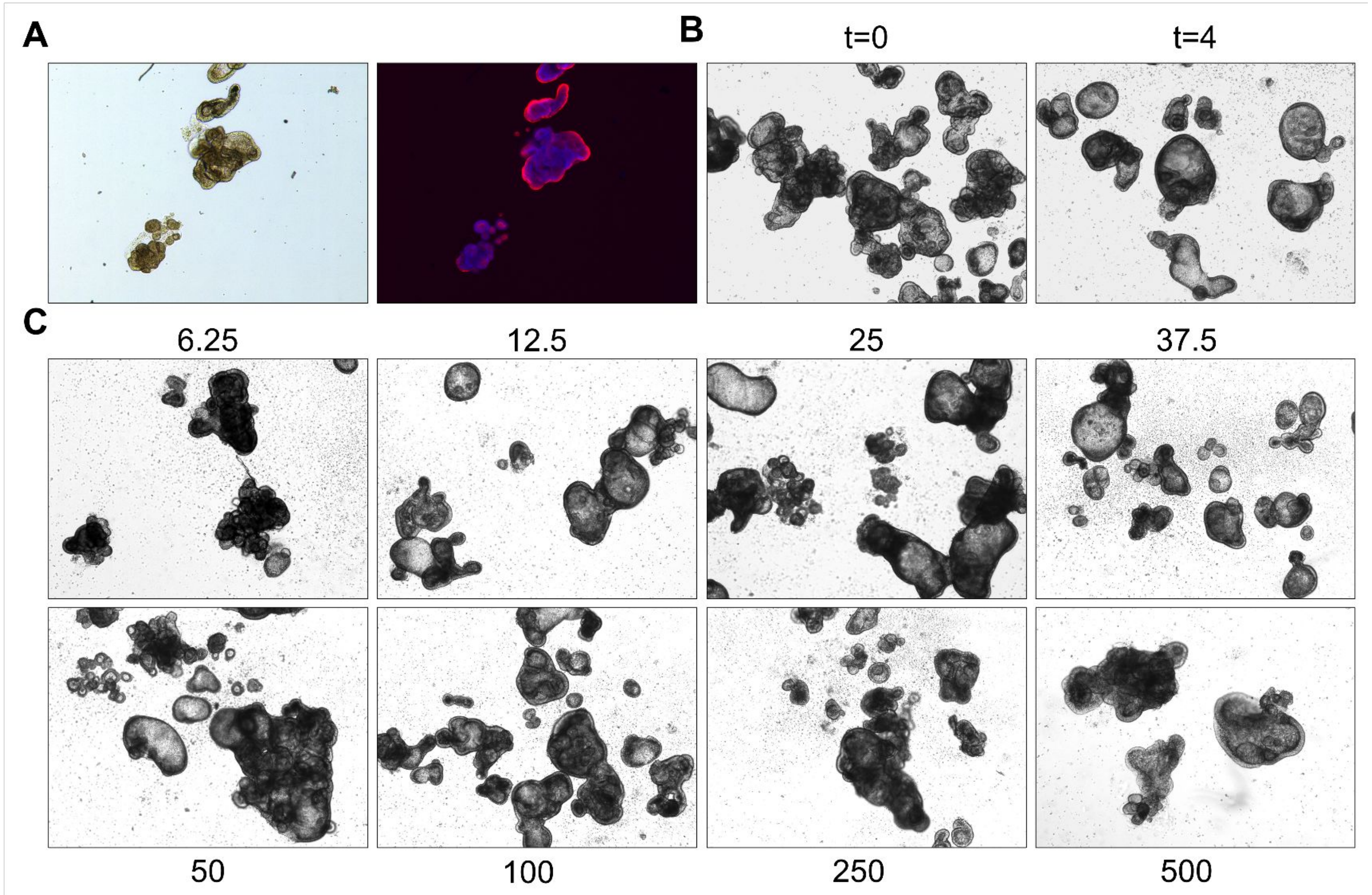

Figure S2. **Microscope pictures of apical-out ileal enteroids after licochalcone A (licoA) exposure**. Panel (A) shows brightfield and fluorescence microscopy pictures of apical-out ileal enteroids, in which nuclei are stained with DAPI (blue) and actin in the microvilli brush border with ActinRed™ 555 (red). (B) shows apical-out enteroids that are not exposed to licoA (negative control) at t = 0 h and t = 4 h, and (C) shows apical-out enteroids that are exposed to different licoA concentrations. Concentrations are shown above and below pictures and are in µg mL^{-1}. All pictures are shown with 4 x magnification.

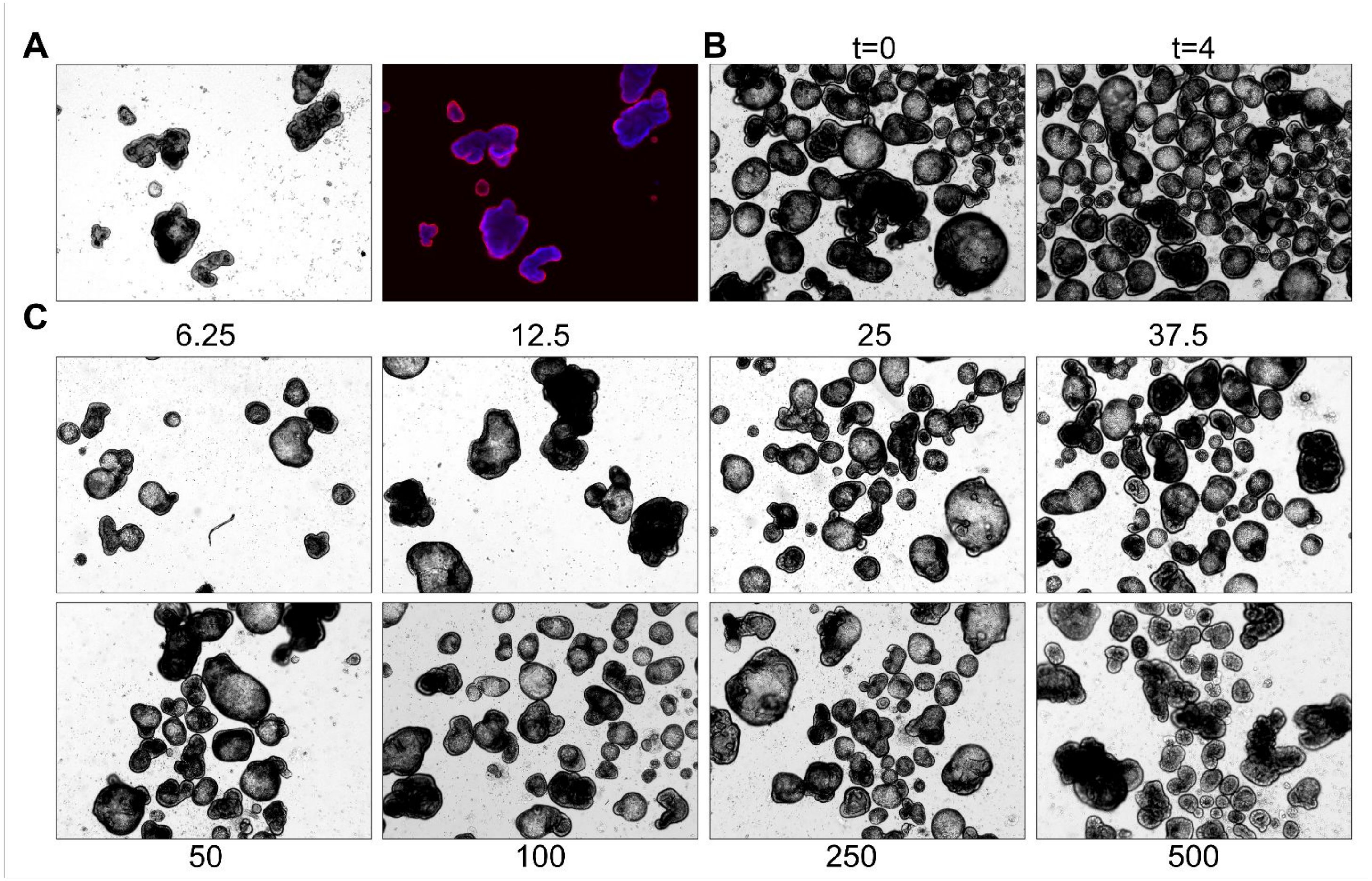

Figure S3. **Microscope pictures of apical-out ileal enteroids after glycycoumarin (glycy) exposure**. Panel (A) shows brightfield and fluorescence microscopy pictures of apical-out ileal enteroids, in which nuclei are stained with DAPI (blue) and actin in the microvilli brush border with ActinRed^{TM} 555 (red). (B) shows apical-out enteroids that are not exposed to glycy (negative control) at t = 0 h and t = 4 h, and (C) shows apical-out enteroids that are exposed to different glycy concentrations. Concentrations are shown above and below pictures and are in μg mL^{-1}. All pictures are shown with 4 x magnification.

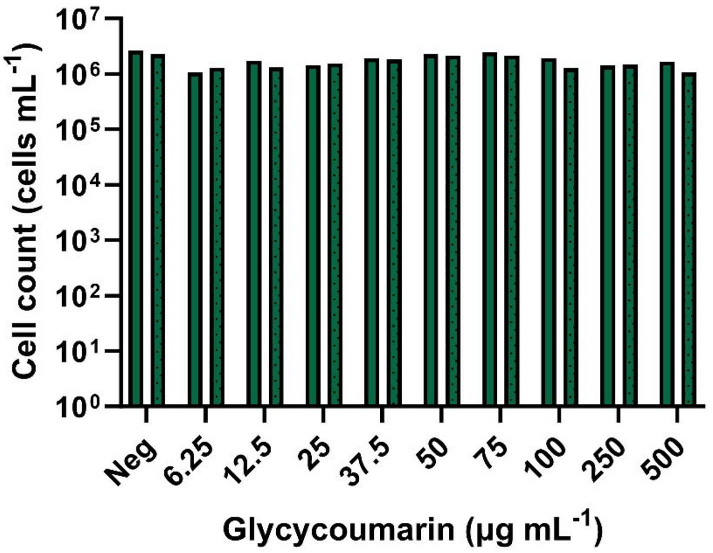

Figure S4. Cell counts of apical-out ileal enteroids. Representative cell count of apical-out ileal enteroids after 4 h glycycoumarin incubation for cytotoxicity (LDH) and cell viability (WST-1) experiments (see **Fig. 3**). Each concentration was tested in duplicate (solid and dashed bars). Enteroids were dissociated to single cells with TrypLE™ Express enzyme reagent (45 min at 37 ˚C) after which they were vigorously pipetted, and counted on a TC20 automated cell counter (BioRad, Hercules, CA, USA).

Table S1. (Tentative) identification of glabridin, licochalcone A, and glycycoumarin and biotransformation products in ileal enteroids. Identification of parent compounds and their biotransformation products in apical-out and apical-in ileal enteroids after 4 h and 24 h exposure, with spectroscopic and spectrometric data obtained in negative ionization (NI) and positive ionization (PI) mode by RP-UHPLC-PDA-ESI-IT-MS^n.

Identified compound	Rt[a] (min)	UV_{max}[b] (nm)	$[M-H]^-$ (*m/z*)	MS^2 NI mode *m/z* (R.A.)[c]	$[M+H]^+$ (*m/z*)	MS^2 PI mode *m/z* (R.A.)	Ref.
**Glabridin**	21.42	282	323	109 (25), 121 (37), 135 (100), 147 (32), 175 (27), 187 (15), 201 (77), 213 (39), 253 (11), 279 (19), 305 (18), 308 (14)	325	123 (39), 189 (100), 203 (20)	
Glabridin-glucuronide	18.18	278	499	175 (53), 323 (100), 437 (21), 481 (37)	501	189 (20), 325 (100), 367 (16), 465 (22), 483 (88)	3
**Licochalcone A**	20.27, 19.70	314, **378**	337	217 (10), 229 (11), 243 (61), 268 (47), 305 (100), 306 (22), 307 (17)	339	121 (31), 219 (11), 245 (22), 271 (33), 297 (100), 298 (23)	
Licochalcone A-glucuronide	14.85, 14.93, 16.53	358, **366**, 382	513	337 (100)	515	339 (100)	
**Glycycoumarin**	18.99	350	367	284 (12), 297 (37), 309 (100), 31 (16), 352 (16)	369	285 (45), 313 (100), 314 (17), 327 (15), 341 (12)	
Glycycoumarin-glucuronide	14.05	346	543	367 (100)	545	369 (100)	4

[a] Retention time in UV; [b] Bold numbers indicate the main UV absorbance peak; [c] R.A. = relative abundance.

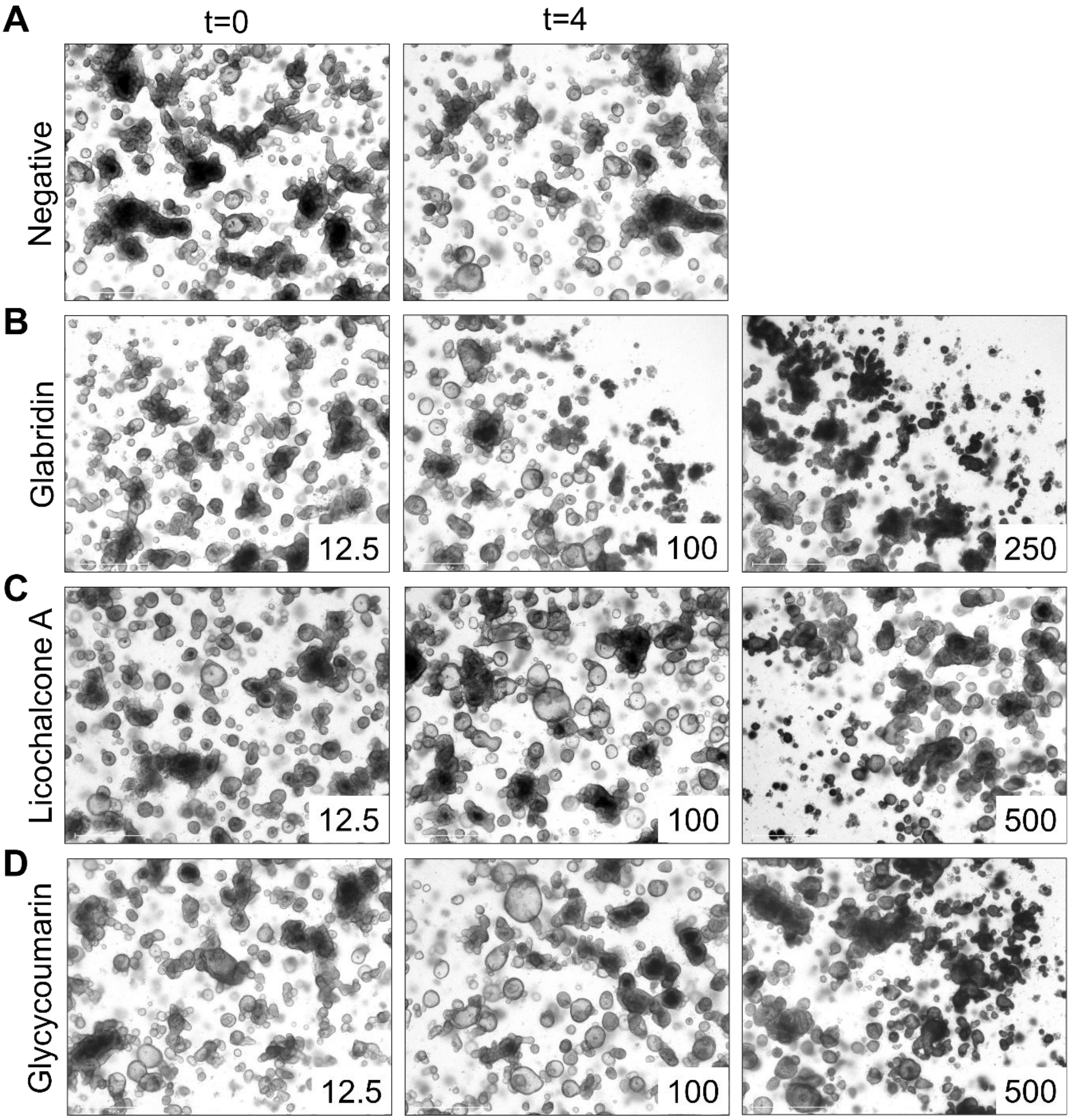

Figure S5. **Microscope pictures of apical-in ileal enteroids after 4 h glabridin (glab), licochalcone A (licoA), and glycycoumarin (glycy) exposure**. Panel (A) shows brightfield microscopy pictures of apical-in ileal enteroids in medium (ODM+DAPT) at t = 0 h and t = 4 h, and panels (B), (C), and (D) show brightfield microscopy pictures of apical-in ileal enteroids after 4 h exposure to different concentrations (in µg mL^{-1}, shown in red) glab, licoA, and glycy, respectively. All pictures are shown with 4 x magnification.

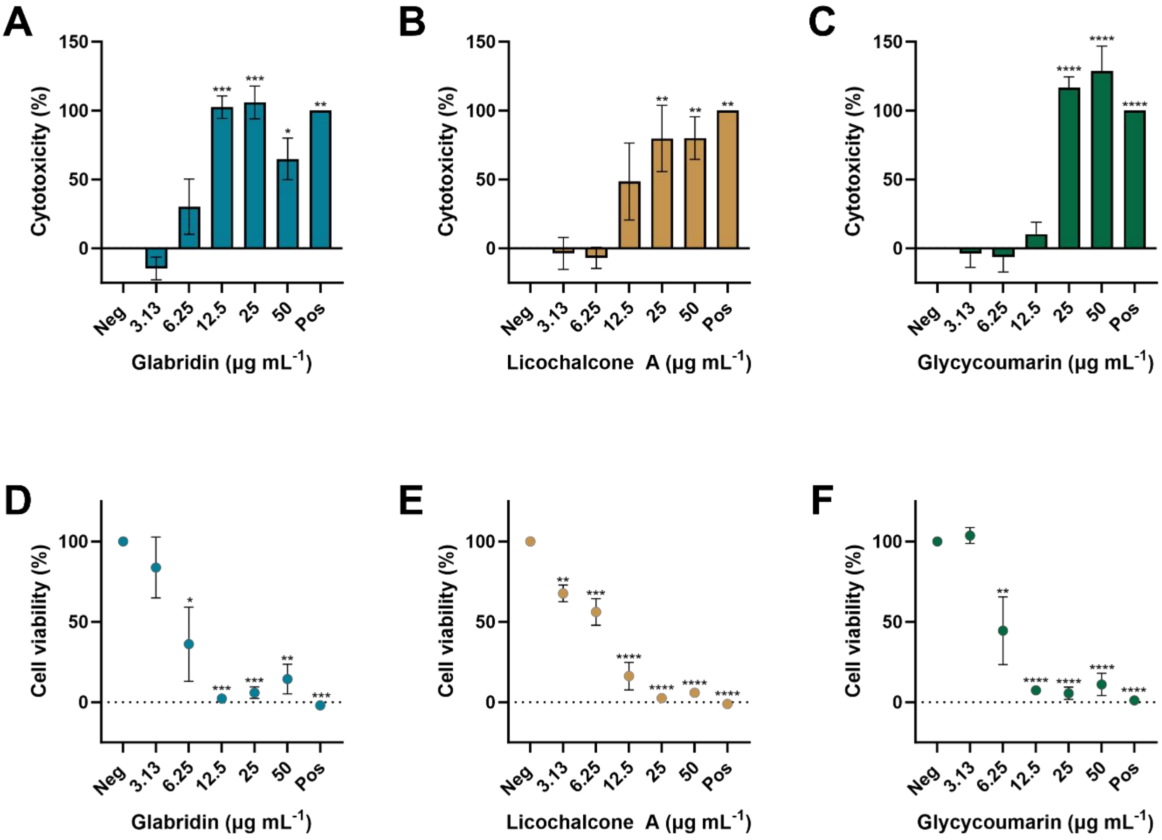

Figure S6. Cytotoxicity and effects on cell viability of glabridin (glab), licochalcone A (licoA), and glycycoumarin (glycy) on proliferating Caco-2 cells. Panels A-C show cytotoxicity and D-F effects on cell viability after exposure to glab, licoA, and glycy on proliferating Caco-2 cells. For the statistics, data are compared to the negative control (neg) and expressed as the mean ± SEM of three biological replicates, measured in triplicate. $^{*}p < 0.05$, $^{**}p < 0.01$, $^{***}p < 0.001$, and $^{****}p < 0.0001$. Neg = negative control and pos = positive control (proliferating Caco-2 cells treated with 1 % Triton™ X-100).

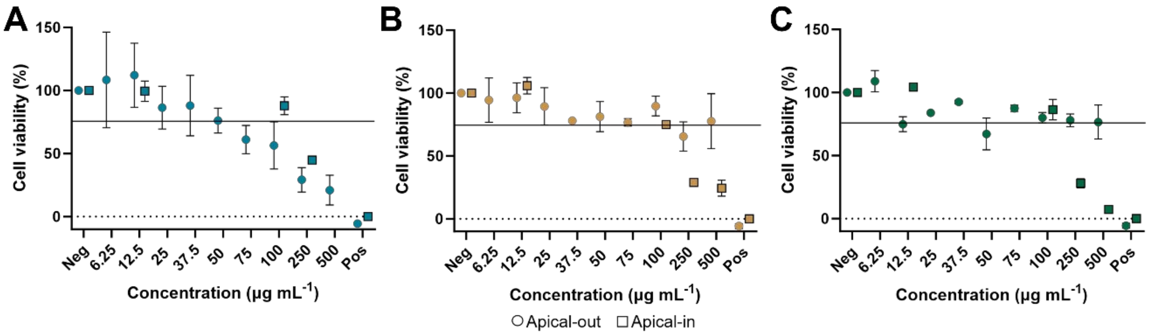

Figure S7. Effects on cell viability in human apical-out (circles) and apical-in (squares) enteroids after (A) glabridin, (B) licochalcone A, and (C) glycycoumarin exposure. Cell viability was determined with WST-1 in µg mL^{-1} after 4 h exposure. A cut-off threshold of 25 % in cell viability compared to the negative control (enteroids in ODM) was used. Neg = negative control and pos = positive control (enteroids treated with 1 % Triton™ X-100).

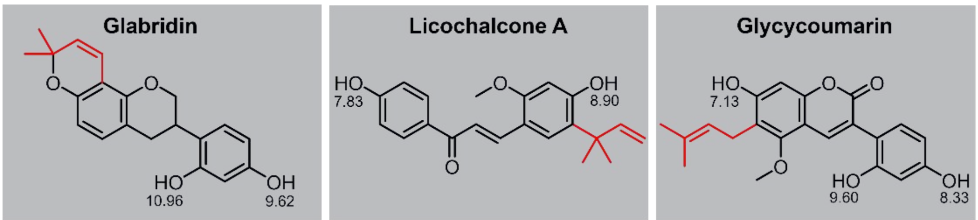

	Glabridin	Licochalcone A	Glycycoumarin
**Strongest acidic pKa**	9.62	7.83	7.13
**% dissociated**	0.6	27.7	67.5
**LogD**	4.09	4.67	3.63

Figure S8. Molecular characteristics of glabridin, licochalcone A, and glycycoumarin. The molecular structures of glab, licoA, and glycy are shown together with their pKa values (shown below the hydroxyl groups), their strongest acidic pKa values, and their percentage dissociation and logD at pH 7.4 Prenyl groups are highlighted in red. pKa and LogD values were calculated with Marvin JS (Chemaxon).

Table S2. Overview of the experimental conditions for exposure experiments with glabridin, licochalcone A, and glycycoumarin in the different cell models.

	**Apical-out enteroids**	**Apical-in enteroids**	**Proliferating Caco-2**	**Differentiated Caco-2**[2]
Growth conditions				
Medium	OGM	OGM	DMEM + FCS	DMEM + FCS
Time (d)	7 – 10	7 – 10	2	21
Differentiation conditions				
Medium	ODM + 5 µM DAPT	ODM + 5 µM DAPT	n.a.[(a)]	n.a.
Time (d)	3[(b)]	3	n.a.	n.a.
Exposure conditions				
Medium	ODM+ 5 µM DAPT	ODM+ 5 µM DAPT	DMEM − FCS	DMEM − FCS
Incubation time (h)	0, 4, 24	0, 4, 24	4	4
Concentration range (µg mL^{-1})	6.25, 12.5, 25, 37.5, 50, 75, 100, 250, 500	12.5, 100, 250, 500	3.13, 6.25, 12.5, 25, 50	3.13, 6.25, 12.5, 25, 50, 100

[(a)]n.a. = Not applicable, [(b)]Apical-out enteroids were stimulated with prenylated phenolics three days after MG removal. OGM = organoid growth medium, ODM = organoid differentiation medium, DAPT = *N*-[2S-(3,5-difluorophenyl)acetyl]-*L*-alanyl-2-phenyl-1,1-dimethylethylester glycine.

References

1. van Dinteren S, Araya-Cloutier C, Robaczewska E, et al. Switching the polarity of mouse enteroids affects the epithelial interplay with prenylated phenolics from licorice (*Glycyrrhiza*) roots. *Food Funct*. 2024;
2. van Dinteren S, Meijerink J, Witkamp R, van Ieperen B, Vincken J-P, Araya-Cloutier C. Valorisation of liquorice (*Glycyrrhiza*) roots: antimicrobial activity and cytotoxicity of prenylated (iso)flavonoids and chalcones from liquorice spent (*G. glabra, G. inflata*, and *G. uralensis*). *Food Funct*. Nov 15 2022;doi:10.1039/d2fo02197h
3. van de Schans MGM, Bovee TFH, Stoopen GM, Lorist M, Gruppen H, Vincken JP. Prenylation and Backbone Structure of Flavonoids and Isoflavonoids from Licorice and Hop Influence Their Phase I and II Metabolism. *J Agric Food Chem*. Dec 16 2015;63(49):10628-10640. doi:10.1021/acs.jafc.5b04703
4. Wang Q, Qiao X, Liu CF, et al. Metabolites identification of glycycoumarin, a major bioactive coumarin from licorice in rats. *J Pharm Biomed Anal*. Sep 2014;98:287-295. doi:10.1016/j.jpba.2014.06.001
